# Climate-dependent plant responses to earthworms in two land-use types

**DOI:** 10.1007/s00442-023-05493-9

**Published:** 2023-12-26

**Authors:** Qun Liu, Nico Eisenhauer, Stefan Scheu, Gerrit Angst, Miriam Bücker, Yuanyuan Huang, Travis B. Meador, Martin Schädler

**Affiliations:** 1 Department of Community Ecology, Helmholtz-Centre for Environmental Research-UFZ, Halle (Saale), Germany; 2 Johann-Friedrich-Blumenbach Institute of Zoology and Anthropology, University of Göttingen, Göttingen, Germany; 3 German Centre for Integrative Biodiversity Research (iDiv), Halle-Jena-Leipzig, Leipzig, Germany; 4 Institute for Biology, Leipzig University, Leipzig, Germany; 5 Centre of Biodiversity and Sustainable Land Use, University of Göttingen, Göttingen, Germany; 6 Biology Centre of the Czech Academy of Sciences, Institute of Soil Biology and Biogeochemistry, České Budějovice, Czech Republic; 7 Institute of Agricultural and Nutritional Sciences, Martin-Luther-University Halle-Wittenberg, Halle (Saale), Germany; 8 Department of Ecosystem Biology, Faculty of Science, University of South Bohemia, České Budějovice, Czech Republic

**Keywords:** Biomass, Stoichiometry, Climate change, Summer droughts, Plant–soil interactions

## Abstract

Plant nutrient uptake and productivity are driven by a multitude of factors that have been modified by human activities, like climate change and the activity of decomposers. However, interactive effects of climate change and key decomposer groups like earthworms have rarely been studied. In a field microcosm experiment, we investigated the effects of a mean future climate scenario with warming (+ 0.50 °C to + 0.62 °C) and altered precipitation (+ 10% in spring and autumn, − 20% in summer) and earthworms (anecic—two *Lumbricus terrestris*, endogeic—four *Allolobophora chlorotica* and both together within 10 cm diameter tubes) on plant biomass and stoichiometry in two land-use types (intensively used meadow and conventional farming). We found little evidence for earthworm effects on aboveground biomass. However, future climate increased above- (+40.9%) and belowground biomass (+44.7%) of grass communities, which was mainly driven by production of the dominant *Festulolium* species during non-summer drought periods, but decreased the aboveground biomass (− 36.9%) of winter wheat. Projected climate change and earthworms interactively affected the N content and C:N ratio of grasses. Earthworms enhanced the N content (+1.2%) thereby decreasing the C:N ratio (− 4.1%) in grasses, but only under ambient climate conditions. The future climate treatment generally decreased the N content of grasses (aboveground: − 1.1%, belowground: − 0.15%) and winter wheat (− 0.14%), resulting in an increase in C:N ratio of grasses (aboveground: + 4.2%, belowground: +6.3%) and wheat (+5.9%). Our results suggest that climate change diminishes the positive effects of earthworms on plant nutrient uptakes due to soil water deficit, especially during summer drought.

**Supplementary Information:**

The online version contains supplementary material available at 10.1007/s00442-023-05493-9.

## Introduction

Anthropogenic climate change is affecting terrestrial ecosystems and ecosystem functions worldwide (Sala et al. 2000; Thakur et al. 2022), and may change biogeochemical cycles of key elements (Yue et al. 2017). As a pervasive threat to ecosystems, climate change with warming and altered precipitation patterns (precipitation decreases in summer, hereafter called ‘summer drought’, and precipitation increases in spring and fall) are the most dominant factors affecting plant productivity and stoichiometry in Central Europe and other regions of the world (Wu et al. 2011; Franklin et al. 2016; Yue et al. 2017). According to regional climate change projections, Germany will face higher temperatures and an elevated risk of summer drought (Görgen et al. 2010). Summer droughts inhibit terrestrial plant growth and decrease plant biomass across habitats (Gherardi and Sala 2019; Meng et al. 2019; Song et al. 2019), while increased precipitation and warming mostly have a neutral or positive influence on plant growth and biomass (Gherardi and Sala 2019; Song et al. 2019; Shi et al. 2022). More specifically, drought stress can reduce plant nutrient uptake by decreasing the nutrient supply derived from mineralization (Fierer and Schimel 2002; Schimel et al. 2007), impeding nutrient diffusion and mass flow in the soil (Lambers et al. 2008), and hampering nutrient transport within the plant (Bassirirad 2000; Kano-Nakata et al. 2011). Warming-induced drought and summer drought affect plant stoichiometry by inhibiting N and P uptake (Viciedo et al. 2019; Yan et al. 2022), ultimately leading to higher C:N and C:P ratios in plants. In general, higher C:N and C:P ratios in plants indicate lower nutritional quality, decomposability, and palatability of plants to herbivores (Ayres et al. 2009; Han et al. 2011), ultimately limiting nutrient cycling. An increase in precipitation provides higher soil moisture, which promotes plant nutrient uptake and reduces plant C:N ratios (Shi et al. 2022). As nutrients are key to plant growth, the question of how climate change affects the balance of plant nutrients is of great importance.

However, interactive effects of warming and altered precipitation can either amplify or dampen the positive or negative effects of each on plant biomass and stoichiometry (Wu et al. 2011; Yue et al. 2017; Wilschut et al. 2022). For instance, Viciedo et al. (2019) found that warming only increased plant biomass under well-irrigated conditions. However, recent meta-analyses indicated that the interactive effects of warming and precipitation extremes on above- and belowground biomass are small (Wu et al. 2011; Song et al. 2019), this may be due to limited field studies that manipulate both factors. Thus, predicting the effects of multiple climate change factors on plant growth and stoichiometry is still challenging. Moreover, climate change effects might further be modulated by biotic interactions, such as the activity of soil organisms that are critical for decomposition processes and nutrient cycling (Bardgett and van der Putten 2014).

Plant growth and stoichiometry have been reported to be strongly driven by soil fauna (Scheu 2003; Brown et al. 2004; Wardle et al. 2004). As ecosystem engineers, earthworm activities, such as feeding, burrowing, and casting, influence nutrient cycling, soil carbon storage and water infiltration, and thereby ecosystem services (Edwards and Bohlen 1996; Scheu 2003; Coleman et al. 2017). Earthworms are also known to have positive effects on plant productivity via several mechanisms (Scheu 2003; Brown et al. 2004). For instance, earthworms may enhance the competitive strength of grasses over forbs and legumes by increasing N mineralization rates and availability to plants (Wurst et al. 2005; Eisenhauer and Scheu 2008; Craven et al. 2017). A recent meta-analysis showed that earthworms increased crop yield by 25% and aboveground biomass by 23% in agroecosystems mainly through increased N availability (van Groenigen et al. 2014). Moreover, the response of plant growth to earthworms may also depend on their ecological strategy (Lavelle et al. 1998; Eisenhauer and Eisenhauer 2020): epigeic and anecic earthworms feeding on soil surface litter play a key role in enhancing the accessibility of litter material to other organisms by litter breakdown and fragmentation. Anecic earthworms produce permanent vertical burrows, which improve soil water infiltration and oxygen availability to plant roots. Endogeic earthworms live in the top soil layers and form non-permanent horizontal burrows and feed on soil organic matter enhancing soil nutrient availability for plants. These differences in life history traits, nutrition and ecosystem effects may lead to variable plant responses. For example, Laossi et al. (2009b) found that anecic earthworms (*Lumbricus terrestris*) increased plant biomass, while endogeic earthworms (*Aporrectodea caliginosa*) only affected the allocation of N to roots and shoots by increasing the N content of the aboveground system. However, most of the studies focused on the effects of individual ecological groups, and few studies have tested the interactive effects of these groups on plant performance (Laossi et al. , 2009a; b).

Moreover, earthworms may exacerbate the detrimental effects of warming-induced water stress on plant growth by decreasing soil water content, as their burrows represent preferential flow paths (Shipitalo et al. 2004; Eisenhauer et al. 2012). However, Hodson et al. (2023) revealed that the presence of earthworms mitigated the impact of drought on wheat biomass, and this effect was likely to be linked to changes in soil microbiota. Thus, the interacting effects of earthworms and climate change are hard to predict and may further depend on the environmental context, such as plant communities and land-use type.

Plant responses to climate change and earthworms can vary widely among plant species due to differences in growth strategies and plant competition. For instance, *Lolium perenne*, a widely grown forage grass species in the temperate zone, is susceptible to drought stress due to its shallow root system (Sampoux et al. 2013; Bothe et al. 2018). However, *Festulolium*, the interspecies hybrid between ryegrass (*Lolium*) and fescue (*Festuca*) species, is more productive and resilient to abiotic and biotic stress (Humphreys et al. 2006, 2014). Similarly, the effects of earthworms on plant growth can be species-specific (Schmidt and Curry 1999). Additionally, the meta-analysis of van Groenigen et al. (2014) indicated that earthworm presence increased aboveground biomass of crops (+31%) and grasses in pasture (+24%) differently. These variations underscore the importance of considering plant diversity and species-specific responses in predicting and managing the impacts of climate change and soil biota on ecosystem functioning.

We performed a microcosm experiment to test the effects of climate change, including warming and altered precipitation patterns, and earthworms belonging to two functional groups (endogeic: Acl—*Allolobophora chlorotica* and anecic: Lte—*Lumbricus terrestris*) on plant growth and stoichiometry in two contrasting land-use types (four forage grass species in an intensively-used meadow, *Lolium perenne*, *Festulolium*, *Dactylis glomerata*, and *Poa pratensis*; and winter wheat in conventional farming). A previous study (Singh et al. 2019) found that endogeic and anecic earthworms dominate the earthworm communities at our experimental site. Therefore, in our study, we specifically collected and analyzed the main representatives of these ecological groups. We hypothesized that (1) future climate (warming and changed precipitation pattern) affects plant biomass and plant N content, with negative effects on plant growth during summer drought; (2) earthworms increase plant biomass and decrease plant C:N ratio via increasing N uptake, with the response of plants differing between anecic and endogeic earthworms; (3) future climate offsets earthworm effects on plant biomass and nutrient uptake; (4) the effects of climate change and earthworms differ between plant species.

## Materials and methods

### Study site

The study was conducted in the Global Change Experimental Facility (GCEF), Bad Lauchstädt, Germany (51° 23ʹ 30N, 11° 52ʹ 49E, 116 m a.s.l.), which is a large field research platform of the Helmholtz-Centre for Environmental Research (UFZ) and was established in 2013 (Schädler et al. 2019). The site is characterized by a sub-continental, temperate climate. Mean annual precipitation is 498 mm (1896–2013), and mean temperature is 8.9 °C (1896–2013). The soil is classified as Haplic Chernozem (around 70% silt and 20% clay content). The upper soil (0–15 cm) pH ranges from 5.8 to 7.5, and contains 1.71–2.09% total C and 0.15–0.18% total N (Schädler et al. 2019). Mean soil bulk density of the experimental site and the used soil in the microcosms was 1.4 g cm^−3^.

### Experimental set-up

The GCEF platform was arranged in a split-plot design to investigate the effects of future climate change, including warming and altered precipitation patterns, on ecosystem processes under different land-use types (Schädler et al. 2019). Ten (80 × 24 m) main plots were randomly assigned to one of two climate treatments (ambient vs. future). Each main plot was divided into five (16 × 24 m) subplots, which were randomly assigned to one of five land-use types (conventional farming; organic farming; intensively used meadow; extensively used meadow; extensively used pasture).

In future climate treatment, the mean daily air temperature at 5 cm height is increased by 0.55 °C, and the mean daily soil temperature at 1 and 15 cm depth is increased by 0.62 and 0.50 °C, respectively (Schädler et al. 2019). The amount of precipitation in future climate treatment is 110% of the ambient rainfall in spring (March–May) and autumn (September–November), and ~ 80% of the ambient rainfall in summer (June–August).

Our study focused on the effects of climate change and earthworms on four grass species (20% *Lolium perenne*, 50% *Festulolium*, 20% *Dactylis glomerata*, 10% *Poa pratensis*) in the intensively-used meadow and winter wheat (RGT Reform) in conventional farming. For further details on the GCEF design and land-use management regime, see M&M in SI, Fig. S1, and Schädler et al. (2019).

On 19-Oct-2020, 80 experimental microcosms consisting of PVC tubes (four tubes per plot) were set up in the GCEF. Per plot, four PVC tubes (height 25 cm, inner diameter 10 cm, closed at the bottom by a 100 µm nylon mesh to allow drainage) were buried at ground level and filled top soil with earthworm-free. The soil in all tubes was compacted to simulate the density of field soil. On 23-Oct-2020, plant seeds were sown into the tubes and seed density was controlled according to the common seed quantities in the GCEF.

We collected adult individuals of anecic (Lte) and endogeic earthworms (Acl) from a place nearby the GCEF to establish four earthworm treatments: (1) control without earthworms, (2) two Lte, (3) four Acl and (4) two Lte + four Acl (LA). These densities were chosen based on the assessment of earthworms at the same experimental site as given in Singh et al. (2020). The mean total density of earthworms (all species, including juveniles) was 28 ind. m^−2^ with a maximum of 116 ind. m^−2^. Since earthworms typically show a patchy distribution in the field, it is hard to create realistic mean densities in microcosms with a limited area. As adding only one individual includes the danger of total loss due to mortality or escape, we added two *L. terrestris* and four *A. chlorotica* to account for the differing biomass of both species. Four microcosms in a row at equal intervals next to each other were randomly assigned to one of the four earthworm treatments in conventional farming and intensively used meadow under two climate treatments (ambient vs. future), respectively, resulting in 80 microcosms (= 2 climate treatments × 2 land-use types × 4 earthworm treatments × 5 replicates). On 11-Mar-2021, earthworms were introduced to the respective treatments. Together with the earthworms, 1 g of chopped corn straw was added to each tube as a food source for earthworms and for simulating a shallow litter layer. For further details about the microcosm set-up, see M&M in SI and Fig. S1.

### Plant growth and biomass assessment

From 15-Mar-2021 to 19-July-2021, wheat growth was assessed at regular intervals of 2 to 3 weeks. The average height of the aboveground wheat was measured. The number of wheat leaves and spikes was counted. On 19-Jul-2021, and we harvested the wheat from each tube, then separated it into straw (comprising stalks and leaves), spikes, and roots. Roots were washed using a 2-mm sieve. Shoot, spike, and root material were dried separately for 3–4 days at 60 °C to constant weight in a drying cabinet. Based on dry weight, straw, spike, aboveground (straw + spike biomass), and root biomass of wheat were calculated as gram per square meter.

From 10-Mar-2021 to 17-Jun-2021, grass growth was assessed at regular intervals of 2 to 3 weeks. The maximum height of each grass species aboveground was measured. We simulated the first (05-May), second (17-Jun) and third (14-Jul) mowing by cutting grasses in tubes at 5 cm above the ground, sorted the grasses in each tube according to species, and separated the inflorescences (if present) and shoots of each grass species. We harvested the roots of grasses on 19-Jul. Roots were washed using a 2-mm sieve. The weights of the shoot, inflorescences, and roots dried at 60 °C were used to calculate the shoot, inflorescence, aboveground (shoot + inflorescences biomass) and root biomass of each grass species. The timing of individual tasks in the field experiment is shown in Table S1.

We noted that at least some earthworms survived throughout the experimental period in all replicates, but due to time constraints, we were unable to count the number or determine the mass of earthworms during the final sampling.

### Plant chemical analyses

The grass samples of each species were cut into pieces and ground in a mill. The ground samples were analyzed for total carbon C and total N contents using a C/N analyzer (Vario EL III, Elementar Analysensysteme GmbH Jena). The values were determined according to the response of an Acetanilide standard (10.35% N, 71.09% C); the precision of the C and N measurements was ± 0.1 and ± 0.03%, respectively. Soil sampling and analyses are provided in M&M of the SI.

### Calculations and statistical analyses

For each tube, we calculated the amount of aboveground C and N and C:N ratio at the grass community-level as well as for wheat. We defined a community as all species co-occurring within a tube. For each grassland tube, the aboveground community mean C and N contents ([C]com, [N]com) of grasses were calculated as follows (Tang et al. 2018):

$$\left[C or N\right]com=\frac{{\sum }_{i=1}^{s}(\left[C or N\right]i\times Bi)}{{\sum }_{i=1}^{s}\left(Bi\right)}$$

where [C or N]*i* is the content of aboveground C or N (%) of the *i*th grass species, *Bi* is the aboveground biomass (g/m^2^) of the *i*th grass species, and s is the number of species in the community.

For each cropland tube, the aboveground C and N contents ([C], [N]) of wheat were calculated as follows:

$$\left[C or N\right]=\frac{Bsp\times \left[C or N\right]sp+ Bst\times \left[C or N\right]st}{Bsp+Bst}$$

where [C or N]*sp* and [C or N]*st* are the C or N content of the wheat spike and straw; *Bsp* and *Bst* are the aboveground biomass of the wheat spike and straw.

To examine the impact of climate and earthworm treatments on various variables, we employed linear mixed-effects models using the *lme4* (Bates et al. 2015) and *lmerTest* packages (Kuznetsova et al. 2017). For data with repeated measurements of response variables, including grass species-level and community-level measurements (biomass, chemical components, maximum height), as well as the maximum height and leaf numbers of wheat, we included climate (two levels: ambient vs. future), earthworms (*Lte*: with *Lumbricus terrestris*, *Acl*: with *Allolobophora chlorotica*), harvest (or date), and their interactions in the fixed term. Harvest times (or sampling dates) served as repeated factor and were nested in main plot as random effects. For other measurements taken only once, the harvest time variable was excluded from the model. To meet the requirements of parametric statistical tests, plant biomass was log-transformed prior to the analyses. Generalized linear mixed models within the *lme4* package were used to analyze the effects of climate, *Acl*, *Lte*, date, and their interactions on leaf numbers of wheat. Count data (number of wheat leaves) was analyzed assuming Poisson-distributed residuals with a log-link function. We also analyzed the effects of climate, *Acl*, *Lte*, and their interactions on soil moisture, soil C and N contents, and soil C:N ratio using linear mixed-effects models. Tube nested in main plot served as random effect in all the above models. Model assumptions were diagnosed using Shapiro–Wilk test for the normality of model residuals and Levene’s test for the homogeneity of variances. A post-hoc multiple pairwise comparison between the means of earthworm treatments and climate treatments was performed using the TukeyHSD() function (*α* = 0.05). All statistical analyses were carried out in the R statistical software version 4.2.0 (R Core Team, 2022).

## Results

### Effects of climate change and earthworms on plant biomass and leaf number in two land-use types

**Fig. 1:**
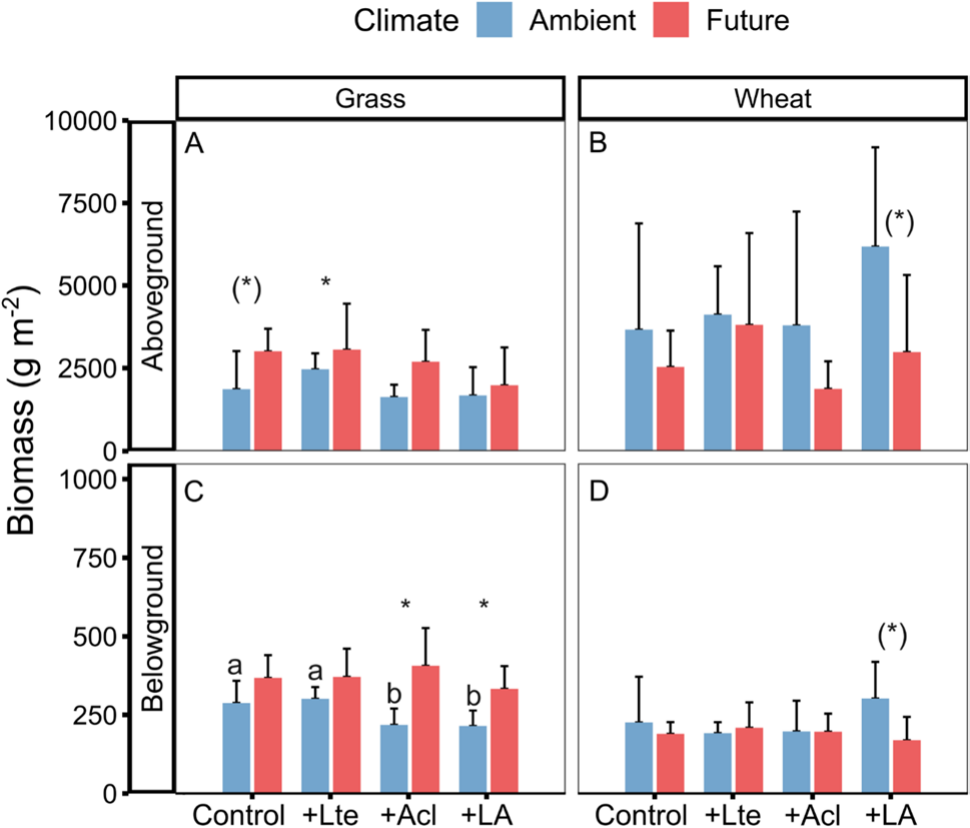
Effects of climate change and earthworms on the total above- and belowground biomass of grass communities and winter wheat (mean + *SD*, *N* = 5). Aboveground biomass of grass communities is the sum of three harvests, while the others are biomass in July. Different lowercase letters denote significant (*P* < 0.05) differences among earthworm treatments (Control: no earthworm, + Lte: only with *Lumbricus terrestris*, + Acl: only with *Allolobophora chlorotica*, + LA: mixed *Lumbricus terrestris* and *Allolobophora chlorotica*) based on post-hoc Tukey’s HSD tests. * and (*) denote significant (*P* < 0.05) and marginal (*P* < 0.10) differences between climate scenarios based on post-hoc Tukey’s HSD tests, respectively

The future climate treatment significantly increased the pooled belowground biomass (+44.7%) and marginally significantly increased the pooled aboveground biomass (+40.9%) across the four grass species (Table 1A, Fig. 1A, C). For wheat, however, the future climate treatment marginally significantly decreased aboveground biomass (− 36.9%), but had no overall effect on belowground biomass (Table 1A, Fig. 1B, D).

**Table 1:** Results (*F*-values and significance levels) from linear mixed-effects models testing the effects of climate (C; ambient vs. future), earthworms (Lte: with *Lumbricus terrestris*, Acl: with *Allolobophora chlorotica*), and their interactions on above- and belowground biomass (A), as well as on carbon (B), nitrogen (C), and C:N ratio (D) of grass communities and winter wheat. Biomass of grasses is the sum of three harvests, while other measurements were from July samples only

	Climate df 1,8	Lte df 1,24	Acl df 1,24	C × Lte df 1,24	C × Acl df 1,24	Lte × Acl df 1,24	C × Lte × Acl df 1,24
(A) Biomass							
Grass above	4.28(*)	0.03	3.37(*)	2.31	0.16	1.97	0.03
Grass below	13.21**	0.40	9.03**	1.11	8.00**	1.49	0.26
Wheat above	4.06(*)	2.48	0.02	0.68	0.92	0.62	0.02
Wheat below	0.78	0.51	0.10	1.71	1.73	0.53	3.1(*)
(B) Carbon							
Grass above	14.21**	1.78	8.37**	2.33	9.38**	0.78	0.64
Grass below	1.77	0.05	0.01	0.22	0.34	0.07	0.01
Wheat above	0.59	0.02	0.10	0.31	3.84(*)	0.00	0.20
Wheat below	0.59	0.08	0.45	1.20	2.30	0.04	0.57
(C) Nitrogen							
Grass above	39.20***	3.71(*)	8.85**	7.83*	6.07*	0.02	8.04**
Grass below	10.52*	1.31	2.64	0.45	0.51	1.21	1.10
Wheat above	5.07(*)	0.05	0.30	0.03	0.14	0.21	0.62
Wheat below	1.55	0.30	1.70	0.42	0.34	0.03	0.01
(D) C:N ratio							
Grass above	28.43***	2.47	7.14*	4.74*	2.48	0.01	8.52**
Grass below	3.98(*)	2.04	1.65	0.70	0.11	2.28	1.64
Wheat above	5.45*	0.06	0.04	0.14	0.29	0.17	0.54
Wheat below	0.40	0.75	1.28	0.10	0.04	0.02	0.20

Numerator degree of freedom and denominator degree of freedom are given in the first row. Significant effects are indicated in bold font, with (*) = *P* < 0.1, * = *P* < 0.05, ** = *P* < 0.01, *** = *P* < 0.001

There was a weak and only marginally significant negative effect of *Acl* on the pooled aboveground biomass of grasses (without: 3063 ± 280 g m^−2^, with: 2848 ± 362 g m^−2^; Table 1). For the pooled belowground biomass, there was a negative effect of *Acl* only under ambient climatic conditions (significant interaction, see Table 1, Fig. 1C).

**Fig. 2:**
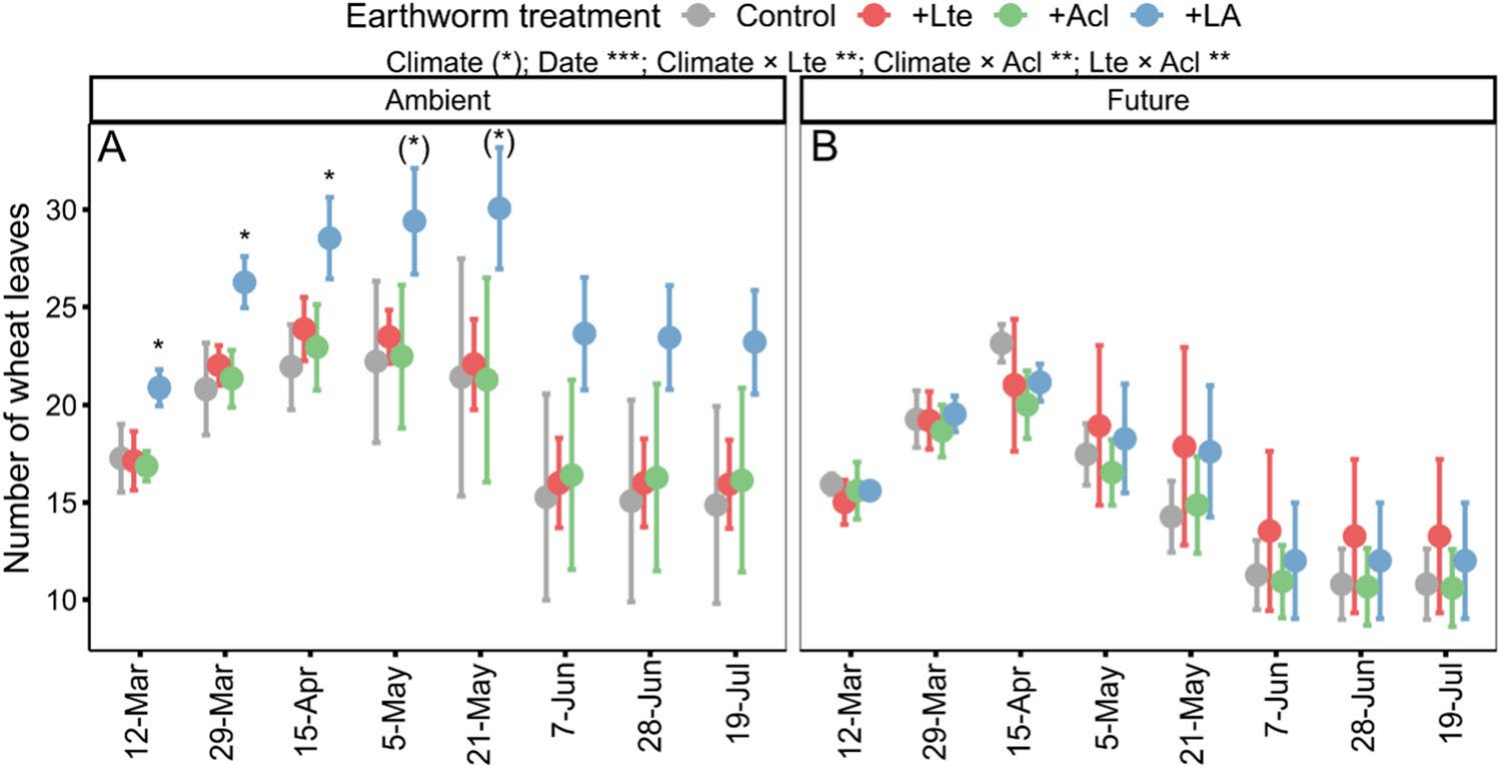
Effects of climate change and earthworms on leaf numbers of wheat (mean + *SD*,* N* = 5). Asterisks and (*) denote significant (*P < 0.05, **P < 0.01, ***P < 0.001) and marginal (*P* < 0.10) effects of climate, sampling date, earthworms (Lte: with *Lumbricus terrestris*, Acl: with *Allolobophora chlorotica*), and their interaction based on generalized linear mixed models, as well as significant and marginal differences among earthworm treatments (Control: no earthworm, + Lte: only with *Lumbricus terrestris*, + Acl: only with *Allolobophora chlorotica*, + LA: mixed *Lumbricus terrestris* and *Allolobophora chlorotica*) based on post-hoc Tukey’s HSD tests, respectively

The combined earthworm (+ LA) treatment significantly increased the leaf numbers of wheat under the ambient climate (Fig. 2A; d.f. = 3, *χ*^2^ = 62.50, *P* < 0.001), but earthworm effects diminished under the future climate (Fig. 2B; d.f. = 3, *χ*^2^ = 4.57, *P* = 0.21). The future climate tended to reduce leaf numbers of wheat during the whole wheat growth process (Fig. 2).

### Effects of climate change and earthworms on plant C and N content in two land-use types

**Fig. 3:**
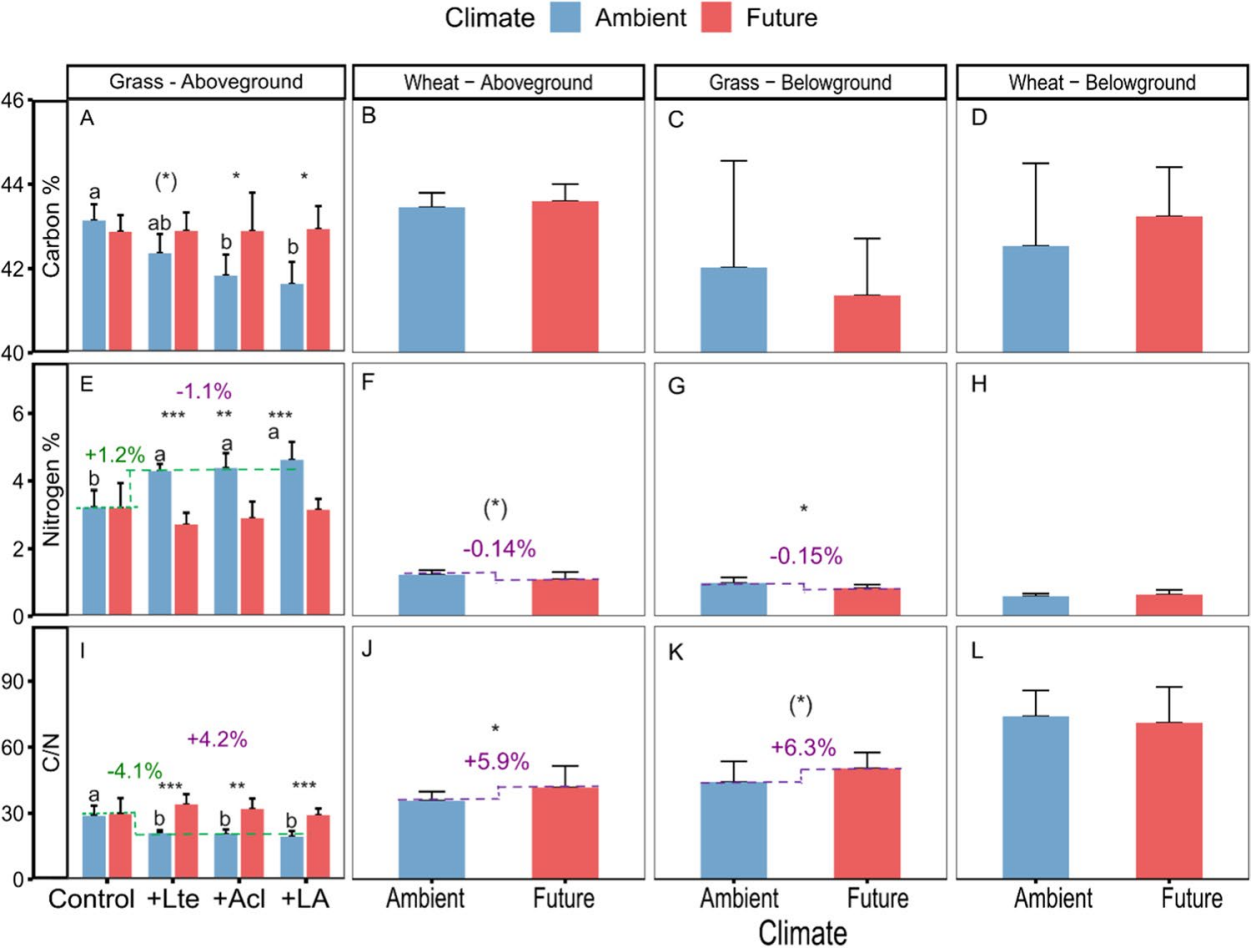
Effects of climate change and earthworms on carbon content, nitrogen content, and C:N ratio in July of above- and belowground grass communities (mean + *SD*,* N* = 5) and winter wheat (mean + *SD*,* N* = 20). Since there were no significant effects of earthworm treatment on C:N ratios in winter wheat and belowground grass communities, the corresponding earthworm treatments are pooled and not shown. Green values are the differences observed between treatments with the presence and absence of earthworms. Purple values are the differences observed between future and ambient climate treatments. Different lowercase letters denote significant (*P* < 0.05) differences among earthworm treatments (Control: no earthworm, + Lte: only with *Lumbricus terrestris*, + Acl: only with *Allolobophora chlorotica*, + LA: mixed *Lumbricus terrestris* and *Allolobophora chlorotica*) based on post-hoc Tukey’s HSD tests. Asterisks and (*) denote significant (*P < 0.05, **P < 0.01, ***P < 0.001) and marginal (*P* < 0.10) differences between climate scenarios based on post-hoc Tukey’s HSD tests, respectively

Among three harvests (May, June, July), the effects of climate change and earthworms were strongest in July on the aboveground grass community-level C, N, and C:N ratio (Table S2, Fig. S4). Climate change significantly increased the aboveground C:N ratio of grasses (+4.2%) and wheat (+5.9%) in July, and marginally significantly increased the pooled belowground C:N ratio of grasses (+6.3%) (Table 1D; Fig. 3I–L). This was mainly caused by an increase in the aboveground C content of grasses and a decrease in the above- (− 1.1%) and belowground N content (− 0.15%) of grasses (significant) in July, as well as the aboveground N content of wheat (− 0.14%) (Table 1B–C, Fig. 3A–H).

There was a similar increase in the pooled aboveground N content (+ 1.2%) as well as a decrease in the aboveground C:N ratio (− 4.1%) and C content of grasses in all three earthworm treatments under ambient, but not under future climatic conditions (Table 1B–D, Fig. 3A, E, I). Moreover, earthworm treatments did not significantly affect the C and N content and C:N ratio of aboveground biomass in wheat and belowground biomass in both grasses and wheat (Table 1B–D, Fig. 3B–D, F–H, J–L). Generally, changes in C and N pool paralleled the changes in plant biomass (Table S3, Fig. S5).

### Aboveground biomass and elemental contents of the four grass species

**Fig. 4:**
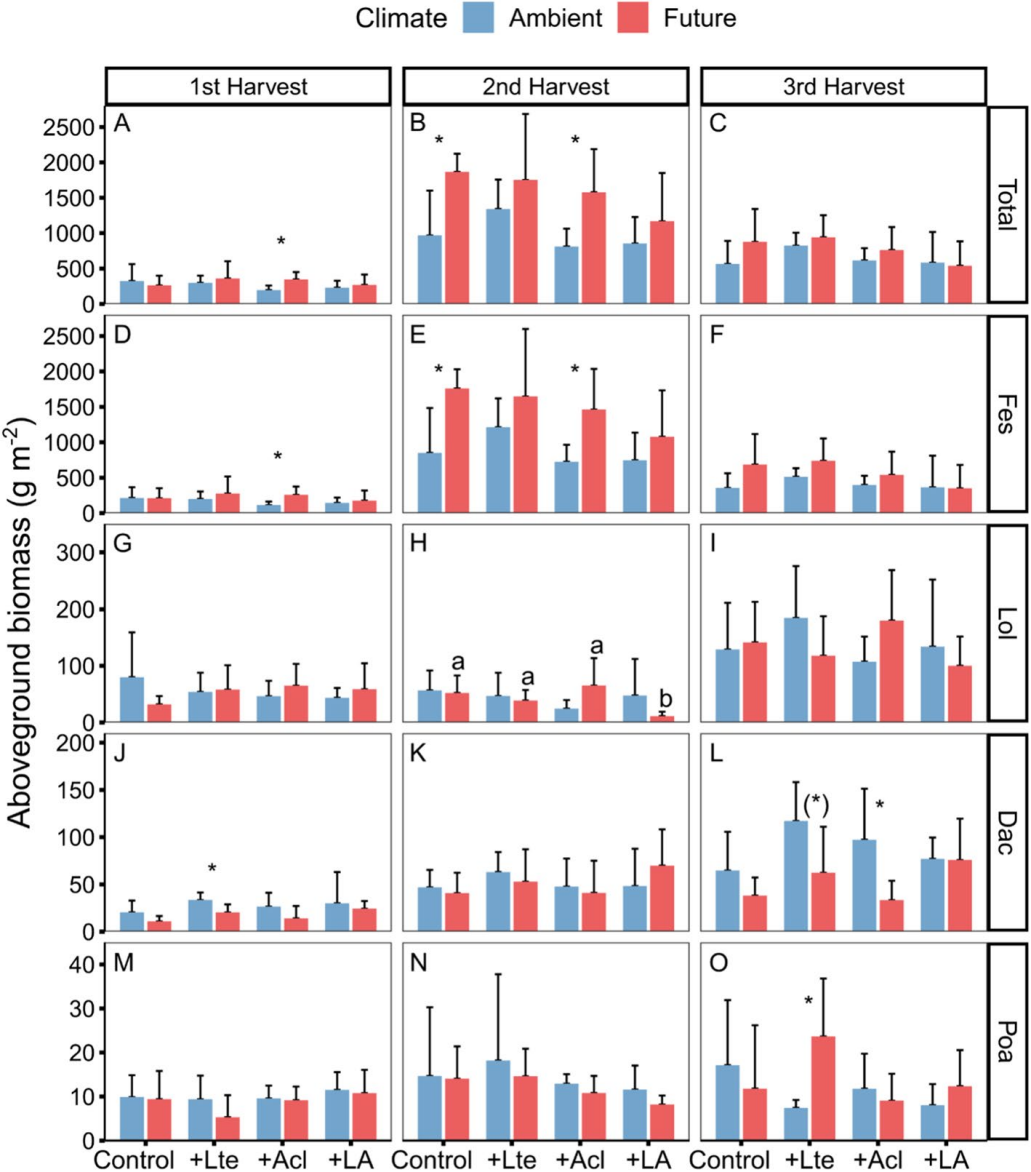
Effects of climate change and earthworms on aboveground biomass of grass at community and species level (Total: total aboveground biomass of four grass species, *Festulolium*, *Lolium perenne*, *Dactylis glomerata*, and *Poa pratense*) in three harvests (1st harvest = 05-May-2021, 2nd harvest = 17-Jun-2021, and 3rd harvest = 14-Jul-2021; mean + *SD*,* N* = 5). Asterisks and (*) denote significant (*P < 0.05, **P < 0.01, ***P < 0.001) and marginal (*P* < 0.10) differences between climate scenarios based on post-hoc Tukey’s HSD tests, respectively. Different lowercase letters denote significant (*P* < 0.05) differences among earthworm treatments (Control: no earthworm, + Lte: only with *Lumbricus terrestris*, + Acl: only with *Allolobophora chlorotica*, + LA: mixed *Lumbricus terrestris* and *Allolobophora chlorotica*) based on post-hoc Tukey’s HSD tests

*Festulolium,* as the dominant grass species, largely determined the overall responses of the grass communities to the experimental treatments across the three harvest dates. While *Festulolium* and the total community reached their highest biomass at the 2nd harvest, the other three grass species did so at the 3rd harvest (Fig. 4). Future climate significantly increased the aboveground biomass of *Festulolium* and, as a result, that of the total grass community, but only at the 2nd harvest (June) (Table 2, Fig. 4A–F). The presence of *Acl* significantly reduced the biomass of *Festulolium* and the total community, especially at peak biomass at the 2nd harvest (Table 2, Fig. 4E).

**Table 2:** ANOVA table (*F*-values and significance levels) from linear mixed-effects model testing the effects of climate (C; ambient vs. future), three harvest times (H; 05-May-2021, 17-Jun-2021, and 14-Jul-2021), earthworms (Lte: with *Lumbricus terrestris*, Acl: with *Allolobophora chlorotica*), and their interactions on aboveground biomass of grass at community and species level (TotalAbove: total aboveground biomass of grass community, Fes: *Festulolium,* Lol: *Lolium perenne*, Dac: *Dactylis glomerata,* Poa: *Poa pratense*)

	df	TotalAbove	Fes	Lol	Dac	Poa
Climate	1,8	7.62*	10.61*	0.06	3.00	0.02
Harvest	2,16	106.18***	107.86***	39.02***	54.14***	2.05
Lte	1,24	0.00	0.01	0.28	5.04*	0.00
Acl	1,24	4.01(*)	3.76(*)	0.46	0.03	2.24
C × H	2,16	9.13**	8.63**	0.00	8.08**	1.08
C × Lte	1,24	1.17	0.98	1.87	0.52	1.06
C × Acl	1,24	0.09	0.25	1.38	0.37	0.14
H × Lte	2,48	0.07	0.04	0.16	1.44	0.02
H × Acl	2,48	3.75*	3.47*	0.15	0.03	2.25
C × H × Lte	2,48	1.97	1.67	4.12*	0.37	3.69*
C × H × Acl	2,48	0.64	0.65	0.49	0.26	0.43
Lte × Acl	1,24	1.19	0.94	0.42	0.52	0.02
C × Lte × Acl	1,24	0.03	0.02	0.96	2.41	0.24
H × Lte × Acl	2,48	0.67	0.57	0.61	1.25	0.70
C × H × Lte × Acl	2,48	0.18	0.15	0.17	2.46(*)	1.01

Numerator degree of freedom and denominator degree of freedom were given in column df. Significant effects are indicated in bold font, with (*) = *P* < 0.1, * = *P* < 0.05, ** = *P* < 0.01, *** = *P* < 0.001

Under future climate conditions, there was a tendency toward a decreased biomass of *L. perenne* in treatments with *Lte* during the 2nd harvest ((Table 2; Fig. 4G–I). *Lte* generally increased the biomass of *D. glomerata* (significant main effect, see Table 2), but this was most pronounced at the 3rd harvest and only under ambient conditions and the absence of *Acl* (4-way interaction, see Table 2, Fig. 4J–L). Likewise, *Acl* increased the biomass of *D. glomerata* at the 3rd harvest only under ambient conditions and in the absence of *Lte*. *Lte* alone significantly increased *P. pratense* aboveground biomass under future climate conditions at the 3rd harvest (Table 2; Fig. 4M–O).

**Fig. 5:**
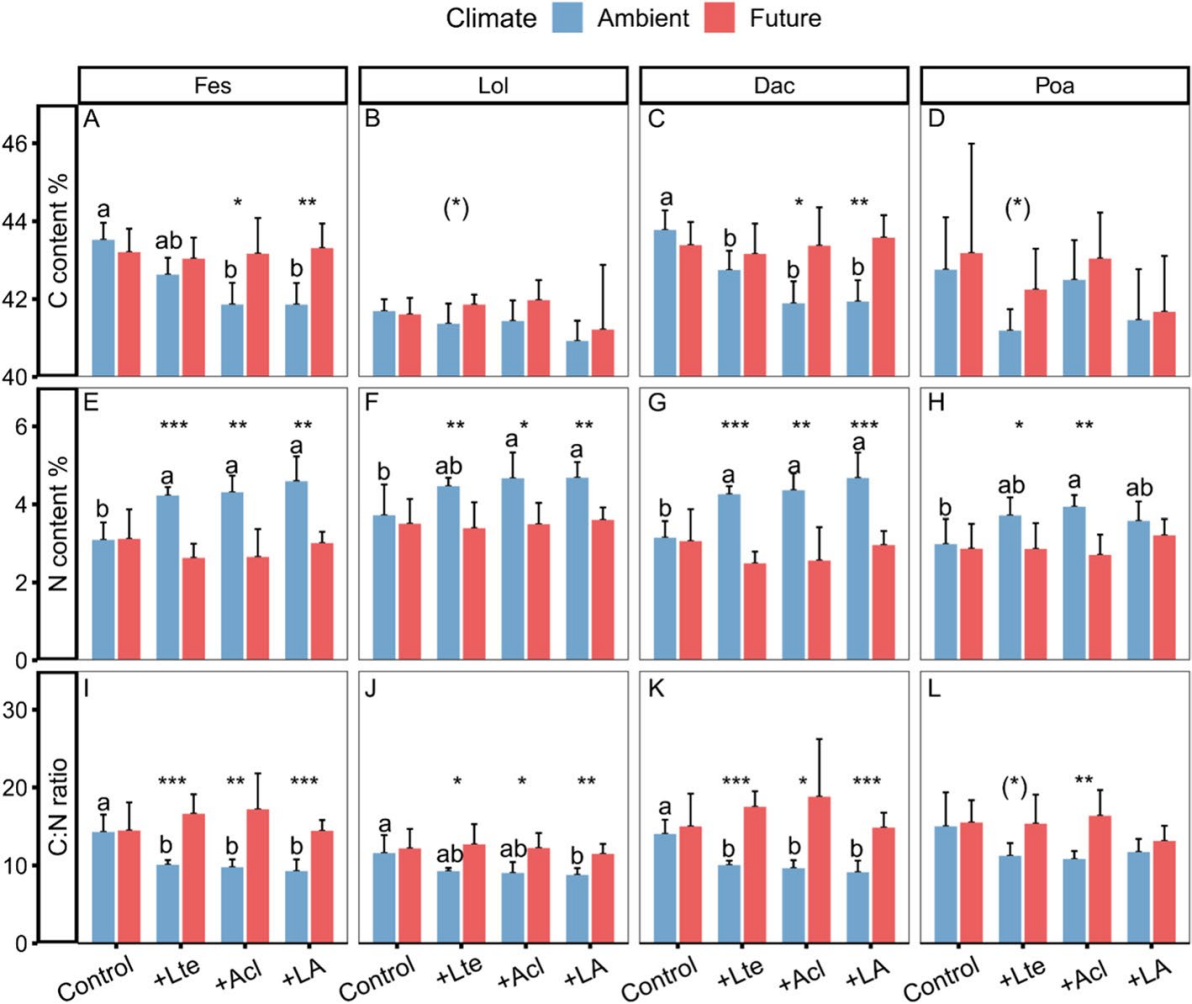
Effects of climate change and earthworms on aboveground plant carbon content, nitrogen content, and C:N ratios of four grass species (Fes: *Festulolium*, Lol: *Lolium perenne*, Dac: *Dactylis glomerata*, Poa: *Poa pratense*) in July (mean + *SD*,* N* = 5). Asterisks and (*) denote significant (*P < 0.05, **P < 0.01, ***P < 0.001) and marginal (*P* < 0.10) differences between climate scenarios based on post-hoc Tukey’s HSD tests, respectively. Different lowercase letters denote significant (*P* < 0.05) differences among earthworm treatments (Control: no earthworm, + Lte: only with *Lumbricus terrestris*, + Acl: only with *Allolobophora chlorotica*, + LA: mixed *Lumbricus terrestris* and *Allolobophora chlorotica*) based on post-hoc Tukey’s HSD tests

Throughout the experiment, C contents in aboveground material of all grass species were highest at the 2nd harvest, whereas N contents constantly increased over time, resulting in a constant decrease in the C:N ratio (Table S4, Fig. S6–S8). Future climate strongly increased the aboveground C:N ratios of *Festulolium*, *L. perenne* and *D. glomerata*, mainly at the final harvest (Fig. 5I–K). This was mainly caused by an increase in the aboveground C contents of *Festulolium* and *D. glomerata,* and the decrease in the aboveground N contents of these three grass species (significant) at the final harvest (Fig. 5A–H). Regarding *P. pratense*, climate change increased the aboveground C:N ratio at the final harvest by decreasing the aboveground N contents (Fig. 5D, H, L).

The C and N contents and C:N ratio of all grass species responded in complex ways to the presence of earthworms depending on harvest date and climate (Table S4, Fig. S6–S8). Earthworms significantly decreased the aboveground C:N ratios of *Festulolium*, *L. perenne* and *D. glomerata* under ambient climate conditions at the final harvest (Fig. 5I–K). The C content was reduced under ambient climate conditions in treatments with *Acl* in *Festulolium* and *D. glomerata* at the final harvest*.* A similar trend occurred for *Lte* and most grass species except for *L. perenne* (Fig. 5A–D)*.* N contents showed a significant increase with earthworms in all four grass species under ambient climate conditions at the final harvest (Fig. 5E–H). However, the C and N contents and C:N ratio did not significantly differ between the + *Lte* and + *Acl* treatments (Fig. 5A–L).

### Soil environmental properties

Climate change significantly decreased soil moisture in conventional farming and tended to decrease in intensively used meadow (Fig. S12A, B). The presence of *Lte* alone increased the soil N content and decreased the soil C:N ratio under ambient conditions (see Fig. S12E, H). However, climate change and earthworms had no effects on the soil C content (Fig. S12C, D).

## Discussion

In our study, we could show that (1) a projected average future climate scenario had a mainly positive effect on the above- and belowground biomass of grasses, but a slightly negative effect on the aboveground biomass of winter wheat in management systems, whose productivity is to a great extent based on periods outside the projected summer drought; (2) warming and summer drought under future climate conditions increased the C:N ratio and decreased the N content of grasses and winter wheat at the final harvest; (3) earthworms had contrasting effects on the stoichiometry of grasses in the different climate treatments, with significantly decreased aboveground C:N ratio and increased aboveground N content of grass at the final harvest only under ambient climate conditions; (4) for winter wheat, only the combined earthworm (+ LA) treatment significantly increased leaf numbers under ambient climate conditions. These results indicate that climate change can cause complex and context-dependent interaction effects with earthworms on plant performance across land-use types.

### Climate change effects

Contrary to our first hypothesis, the simulated future climate, including warming and a changed precipitation pattern, increased grass biomass. This positive effect was mainly driven by the dominant grass *Festulolium*, which reached peak biomass earlier (i.e., under warming and precipitation increase conditions) than the other grass species. This aligns with a previous report by Helgadóttir et al. (2014) that spring growth of *Festulolium* is earlier than that of other perennial grasses. Consequently, the results indicate that biomass production of the studied grassland shifts toward periods with slightly more rainfall and warmer temperatures under future conditions. These results are consistent with those of Viciedo et al. 2019, who also observed that warming increases plant biomass under well-irrigated conditions. However, this was not the case in species that reached peak biomass later (i.e., under warming and summer drought conditions), likely due to drought stress and the competitive advantage of *Festulolium,* which is characterized by high productivity and resilience to both abiotic and biotic stress (Humphreys et al. 2006, 2014). It is therefore likely that future climates would adversely affect grass species that develop later in the year, which were not considered in this experiment.

The future climate only slightly negatively affected the growth of winter wheat, possibly due to alleviating effects of increased precipitation in earlier plant stages and decreased precipitation in later stages. Specifically for winter crops, increased rainfall has also been shown to compensate for negative effects of spring frost (Bai et al. 2022), which may be of increasing relevance under conditions of future warming and advanced plant phenology (Gu et al. 2008). In general, additional precipitation mitigates the yield losses caused by other climate factors by improving soil moisture conditions during the growing season and counteracting warming effects on soil nutrients (Rosenzweig et al. 2002; Shi et al. 2022).

Warming and summer drought in future climate treatment primarily decreased the aboveground N content in grasses and winter wheat, as well as the belowground N content in grasses, leading to higher C:N ratios at the final harvest. This is in accordance with our first hypothesis and other studies reporting negative effects of drought on plant nutrient uptake (Viciedo et al. 2019; Yan et al. 2022). Presumably, soil water deficit decreases the absorption of N from soil due to reduced water potential at the root surface (Bassirirad 2000; Kano-Nakata et al. 2011), reduced nutrient diffusion and mass flow in soil (Lambers et al. 2008), and reduced nutrient supply resulting from decreased soil nitrification and N mineralization rates (Fierer and Schimel 2002; Schimel et al. 2007). Indeed, in our study, soil moisture was decreased by climate change, but without affecting the soil N content. This suggests that the effects of climate change on plant stoichiometry were mainly mediated by soil moisture.

These results demonstrate that land management could mitigate the effects of climate change on plant production by selecting management regimes and cultures, such as full irrigation and appropriate shift of sowing dates, which support plant growth, especially outside of projected summer droughts.

### Earthworm effects

Contradictory to our second hypothesis and previous studies suggesting that earthworms generally improve plant productivity (Wurst et al. 2003; Scheu 2003; Eisenhauer and Scheu 2008), there was insufficient evidence that earthworms generally increase above- and/or belowground biomass of grasses. In the present study, the presence of *Acl* marginally decreased the aboveground biomass and significantly decreased the belowground biomass of the grass community. This may have been due to negative effects of *Acl* on the biomass of the dominant grass species, *Festulolium*, at the 2nd harvest. Moreover, previous studies have demonstrated that earthworms have neutral, positive, or negative effects on individual grass species when competing with other plants (Wurst et al. 2005; Eisenhauer et al. 2009). Therefore, it is plausible that the observed negative effects of *Acl* on *Festulolium* and positive effects of earthworms on *D. glomerata* could be attributed to interspecific competition.

Furthermore, earthworms significantly increased the aboveground N content of grasses under ambient climate conditions, suggesting that earthworms indeed increased N uptake by grasses, but only at the final harvest. Potentially, at earlier stages of harvesting, the soil still contained sufficient amounts of nutrients to meet the plant demands due to the application of fertilizer. Van Groenigen et al. (2014) also concluded that the positive effects of earthworms disappear when soil nitrogen availability is high. Therefore, the effects of earthworms were not significant at the first two harvests. However, at later stages when plant nutrient demand increases, earthworms become more important in supplying nutrients by mobilizing N from organic matter (Edwards and Bohlen 1996; Scheu 2003). This is further supported by the increase in soil N content and decrease in soil C:N ratio observed in the + Lte treatment in this study. Additionally, earthworms can indirectly increase nutrient availability by enhancing soil microbial activity due to feeding, burrowing and casting which may promote the decomposition of organic matter (Blouin et al. 2013). Research on earthworm–microbiome–plant interactions improves our understanding of aboveground-belowground interactions (Jacquiod et al. 2020). However, we did not measure microbial activity or community composition in the current study, and inclusion of these measures in future studies would provide further insights. Nevertheless, it should be noted that earthworms did not have any significant impact on the belowground N content of grasses. This indicates that earthworms might only influence grasses to allocate more N to aboveground, aligning with the findings of Laossi et al. (2009b) about how earthworms affect N allocation in plants. However, this contrasts with the findings of Eisenhauer and Scheu (2008) that additional N resulted in higher biomass production, but constant N concentrations in plants.

Inconsistent with our second hypothesis and the findings of a meta-analysis (van Groenigen et al. 2014), there was a lack of earthworm effects on the biomass and stoichiometry of winter wheat in conventional farming. Potentially, winter wheat growth was not limited by N, likely due to mineral N fertilization, which is applied at rates of 27 kg N ha^−1^ yr^−1^ in this conventional farming practice. Van Groenigen et al. (2014) also pointed out that the high application rate of fertilizers mitigates the effects of earthworms on crop production, especially when application rates exceeded 30 kg N ha^−1^ yr^−1^. Therefore, the contribution of earthworms to supply nutrients by mineralization may have been masked in this land-use type. Additionally, the wheat straw in the present study had low nutrient contents, which may further limit the contribution of earthworms to nutrient availability. Despite the weak earthworm effects on crop growth in present study, it is still important to promote and protect earthworm populations in agricultural systems, as they have been shown repeatedly to play a crucial role in maintaining soil functions, such as nutrient cycling, water infiltration, and soil aggregate and C stability through feeding, burrowing, and casting (Lavelle et al. 1998; Coleman et al. 2017; Angst et al. 2022).

The effects of the *Acl* (endogeic) and *Lte* (anecic) on plant stoichiometry were not significantly different. Moreover, we only found few interaction effects between the two earthworm species, which is consistent with the findings of Laossi et al. (2009a). The reason might be that both types of earthworms can enhance nutrient availability by breaking down organic matter in the soil and releasing nutrients (Lavelle et al. 1998; Eisenhauer and Eisenhauer 2020).

### Interactive effects of climate change and earthworms

Partially supporting our third hypothesis, simulated future climate diminished the effects of earthworms on the N content of grasses. Specifically, earthworms significantly increased the N content of grasses under ambient conditions at the last harvest, leading to a decrease in the C:N ratio of grass shoots. Potentially earthworms enhance the release of N from residues and soil organic matter and indirectly improving N uptake by plants (Scheu 2003; Brown et al. 2004). However, the reduced soil water content under future climate conditions inhibits N absorption by plants from soil by reducing the water potential at the root surface (Bassirirad 2000; Kano-Nakata et al. 2011) as well as reducing nutrient diffusion and mass flow in soil (Lambers et al. 2008). This indicates that soil water deficit is a more critical factor affecting plant nitrogen uptake than earthworms in future climate treatments. Interestingly, there were no significant interactive effects between climate change and earthworms on grass growth at the first two harvests. Only grass biomass increased in future climate treatment. This implies that earthworms did not mitigate the negative effects of the future climate on plant stoichiometry during the summer drought or enhance the positive effects of the future climate on grass biomass outside the summer drought. This finding highlights the need for novel management strategies to alleviate the consequences of climate change.

For winter wheat, however, there were hardly any interactive effects between climate change and earthworms, except that the combined earthworm treatment (+LA) significantly increased leaf numbers during the early growing stage in the ambient but not in future climate. By increasing soil nutrient conditions, earthworms may cause indirect effects, such as increased plant investment into the number of tillers. This is supported by studies on earthworm–plant interactions (Wang et al. 2022). However, the warmer temperatures in future climate could advance crop phenological stages to a cooler period of the growing season, which could lead to an increase in the risk of damage due to spring frost (Gu et al. 2008). This increase in spring frost risk is known to contribute mainly to the reduction of the number of tillers and spikes (Li et al. 2015), potentially diminishing the beneficial effects of earthworms on winter wheat growth under future climate conditions. Our results indicate that fostering earthworms as a management measure alone may not be an effective way to alleviate the effects of climate change on winter wheat. This aligns with the suggestion of Hodson et al. (2023) that improving plant tolerance to drought may be an important strategy, in addition to fostering earthworm numbers.

In general, the grassland community showed somewhat stronger responses to the experimental treatments than wheat. This can be explained by the different life cycles of the target plants. While grasses and wheat were sown at the same time, i.e., in the fall of the previous year, the final harvest was done at the point of wheat maturity. The perennial grasses of the grassland community, however, are perennial species that grow during the whole year, whereas wheat mainly gains biomass in spring. Furthermore, application of fertilizer in spring may have masked the earthworm effects on grass biomass during the earlier stage of this experiment (van Groenigen et al. 2014). During the late stage of this experiment, the growing season for wheat was over, but grass growth continued. Therefore, the effects of earthworm-induced alterations in nutrient availability and summer drought might be more relevant to grasses than to wheat.

Earthworm incubation experiments are widely used to study the linkage between aboveground and belowground processes. Earthworm activity in soil is known to have positive effects on plant productivity via several mechanisms. In this study, we observed the survival of earthworms in all replicates throughout the experimental period, but due to time constraints, we were unable to count the number or determine the mass of earthworms during the final sampling. Thus, we cannot exclude positive effects of earthworms on plant N uptake due to soil nutrient mobilization caused by the decay of earthworm bodies.

## Conclusions

In summary, our study revealed that effects of climate change and earthworms interactively influence the growth of grass but not that of winter wheat. Increased N uptake by grasses due to earthworm presence was diminished under future climate conditions with warming and summer drought. On the other hand, earthworms had little effect on the biomass and stoichiometry of winter wheat, neither under ambient nor future climate conditions. Future climate conditions increased the above- and belowground biomass of grasses, but slightly decreased the aboveground biomass of winter wheat. Our study emphasizes the importance of considering the effects of climate change on plant growth in agro-ecosystems and the limitations of earthworms in mitigating these effects. As a consequence, appropriate measures need to be implemented to both mitigate the causes and consequences of climate change for sustainable management across land-use types.

### Supplementary Information

Below is the link to the electronic supplementary material.

Supplementary file1 (DOCX 4623 KB)

## Data Availability

The datasets used during the current study are available from the corresponding author on reasonable request.

## Code availability

R code will be made available on request.
