# Supplementary material for: Climate-dependent plant responses to earthworms in two land-use types
Source: Oecologia. 2023 Dec 26;204(1):133–46. doi: 10.1007/s00442-023-05493-9 (PMC10830777; doi:10.1007/s00442-023-05493-9)
Supplement: Supplementary file 1 — Supplementary file1 (DOCX 4623 KB) [file 442_2023_5493_MOESM1_ESM.docx]

**Supplementary information**

**Climate-dependent plant responses to earthworms in two land-use types**

Qun Liu^1,2*^, Nico Eisenhauer^3,4^, Stefan Scheu^2,5^, Gerrit Angst^3,4,6^, Miriam Bücker^7^, Yuanyuan Huang^3,4^, Travis B. Meador^6,8^, Martin Schädler^1,3^

**Affiliations:**

^1^ Department of Community Ecology, Helmholtz-Centre for Environmental Research-UFZ, Halle (Saale), Germany

^2^Johann-Friedrich-Blumenbach Institute of Zoology and Anthropology, University of Göttingen, Göttingen, Germany

^3^German Centre for Integrative Biodiversity Research (iDiv), Halle-Jena-Leipzig, Leipzig, Germany

^4^Institute for Biology, Leipzig University, Leipzig, Germany

^5^Centre of Biodiversity and Sustainable Land Use, University of Göttingen, Göttingen, Germany

^6^Biology Centre of the Czech Academy of Sciences, Institute of Soil Biology and Biogeochemistry, České Budějovice, Czech Republic

^7^Institute of Agricultural and Nutritional Sciences, Martin-Luther-University Halle-Wittenberg, Halle (Saale), Germany

^8^Department of Ecosystem Biology, Faculty of Science, University of South Bohemia, České Budějovice, Czech Republic

^*^ **Corresponding author:** Qun Liu (E-mail: liu.qun@ufz.de; Tel: +49 345 55 85 307; Fax: +49 345 55 85 329)

**Materials and methods**

*Experimental set-up*

The GCEF platform was arranged in a split-plot design to investigate the effects of future climate change, including warming and altered precipitation patterns, on ecosystem processes under different land-use types (Schädler et al. 2019). Ten (80 × 24 m) main plots were randomly assigned to one of two climate treatments (ambient vs. future). Each main plot was divided into five (16 × 24 m) subplots, which were randomly assigned to one of five land-use types (CF, conventional farming; OF, organic farming; IM, intensively-used meadow; EM, extensively-used meadow; EP, extensively-used pasture).

The climate treatment was based on projections of different dynamic regional climate models for Central Germany for the years 2070-2100 (www.regionaler-klimaatlas.de). We used 12 climate simulations under four different emission scenarios with three established regional climate models: COSMO-CLM (Rockel et al. 2008), REMO (Jacob and Podzun 1997), and RCAO (Doscher et al. 2002), and used a target average scenario, where mean temperature is projected to increase across all seasons by ~2 ℃ and the amount of precipitation is projected to increase by ~10% in spring (March–May) and autumn (September–November), but to decrease by ~20% in summer (June–August).

All main plots are equipped with steel framework (5.5 m height) to account for possible side effects of the construction itself (Fig. S1). In order to simulate the future climate scenario, half of the main plots are further equipped with mobile roofs, side panels, rain sensors and irrigation system. Roofs and the western and eastern side panels are automatically closed at sunset and open at sunrise to increase night temperature, which resulted in an increase of 0.55℃ of the mean daily air temperature at 5 cm height, as well as 0.62℃ and 0.50℃ of the mean daily soil temperature in 1 cm and 15 cm depth, respectively (Schädler et al. 2019). The precipitation pattern is manipulated by the mobile roofs and irrigating rainwater. The rain sensors associated with the irrigation system are used to regulate the amount of precipitation on the future climate to reach 110% of ambient rainfall in spring (March–May) and autumn (September–November), and ~80% of ambient rainfall in summer (June–August).

Our study focused on the effects of climate change and earthworms on four grass species in the intensively-used meadow and winter wheat (RGT Reform) in conventional farming. Conventional farming is characterized by a 3-year crop rotation of winter rape, winter wheat, and winter barley. Mineral fertilizer and pesticides were applied at least 2 to 3 times per year. The application rates of fertilizer and pesticides during the period of this experiment are provided in Table S1. The intensively-used meadow was seeded with mixture of four forage grasses (20% *Lolium perenne*, 50% *Festulolium*, 20% *Dactylis glomerata*, 10% *Poa pratensis*) and was moderately fertilized and frequently mown (3-4 times per year).

Per plot, four PVC tubes (height 25 cm, inner diameter 10 cm, closed at the bottom by a 100 µm nylon mesh to allow drainage) were filled with top soil. The soil had been taken from the upper 30 cm at the same site six years ago and stored dry in plastic boxes and therefore were earthworm-free. Four tubes per plot were buried at ground level and equipped with a ring of transparent plastic of 25 cm height. Escape of earthworms was further prevented by a hook-and-loop fastener at the inner side of the foil (Lubbers and van Groenigen 2013). On 23-Oct-2020, plant seeds were sown into the tubes. In conventional farming, 5 winter wheat seeds were sown in each tube. Next spring, plant density was adjusted according to the plot conditions outside the tubes to three plants per tube. In the intensively-used meadow, the aim was also to sow a mixture density which is identical to the sowing density on the plots. Taking into account the specific seed weights and the size of the area in the tubes, the grasses of the intensive-used meadow were sown in the following densities per microcosm in adaptation to the seed quantities in the GCEF (Schädler et al. 2019):

• *Festulolium* "Fedoro" (Meadow Scurry): 5 seeds

• *Dactylis glomerata* "Lidacta" (Orchard grass): 6 seeds

• *Poa pratense* "Liblue" (Bluegrass): 7 seeds

• *Lolium perenne* "early Karatos" (Perennial Ryegrass): 2 seeds

• *Lolium perenne* "mid Ashtonhockey" (Perennial Ryegrass): 2 seeds

We collected adult individuals of anecic, *Lumbricus terrestris* (Lte; the average fresh weight of ten randomly selected earthworms with gut content was 1.52 ± 0.22 g), and endogeic earthworms, *Allolobophora chlorotica* (Acl; the average fresh weight of ten randomly selected earthworms with gut content was 0.35 ± 0.04 g) from a place nearby the GCEF to establish four earthworm treatments: (1) control without earthworms, (2) two Lte, (3) four Acl, and (4) two Lte + four Acl (LA). These densities were designed based on the assessment of earthworms at the experimental site by Singh et al. (2020). The mean total density of earthworms (all species, including juveniles) was 28 ind. m^-2^ with a maximum of 116 ind. m^-2^. Since earthworms typically show a patchy distribution in the field, it is hard to create realistic mean densities in microcosms with a limited area. As adding only one individual includes the danger of total loss due to mortality or escape, we added two *L. terrestris* but four *A. chlorotica* to account for the differing biomass of both species. Four microcosms in a row at equal intervals next to each other were randomly assigned to one of four earthworm treatments in conventional farming and intensively-used meadow under two climate treatments (ambient vs. future), respectively, resulting in 80 microcosms (= 2 climate treatment × 2 land-use types × 4 earthworm treatments × 5 replicates). On 11-Mar-2021, earthworms were introduced in the respective treatments. Earthworms were not placed in the tubes at the same time as the plant seeds to prevent the earthworms from undermining or eating the seeds and seedlings (Forey et al. 2011). Together with the earthworms, 1 g of chopped corn straw was added to each tube as a food source for earthworms and for simulating a shallow litter layer. The corn straw come from greenhouse culture. It was labeled with 13C and 15N and has a C content of 38.75% and an N content of 0.8%.

*Soil sampling and properties analyses*

We sampled soil cores with a metal corer (5 cm diameter; 10 cm deep) in 19-Jul-2021 from all microcosms to measure soil moisture, and soil organic C and total N concentrations. Gravimetric soil water content was measured from soil samples for microbial analyses (see Scheu, 1992 for details). Total soil C content and total soil N content were measured using an EA-Isolink CNSOH analyzer (ThermoFisher Scientific, Bremen, Germany). The values were determined according to the response of a Peat Soil Standard (0.3-1.7 mg; N = 23). The precision of the C and N measurements of soil were ± 0.4% and ± 0.15%, respectively. The monthly mean soil moisture and soil temperature in each plot during the period of this experiment were automatically measured by using a high-scalable ad-hoc wireless sensor network.

**References**

Doscher R, Willen U, Jones C, Rutgersson A, Meier HEM, Hansson U, Graham LP (2002) The development of the regional coupled ocean-atmosphere model RCAO. Boreal Environ Res 7:183-192. doi: http://refhub.elsevier.com/S0038-0717(19)30009-4/sref13

Forey E, Barot S, Decaëns T, Langlois E, Laossi K-R, Margerie P, Scheu S, Eisenhauer N (2011) Importance of earthworm–seed interactions for the composition and structure of plant communities: A review. Acta Oecologica 37:594-603. doi: 10.1016/j.actao.2011.03.001

Jacob D, Podzun R (1997) Sensitivity studies with the regional climate model REMO. Meteorol Atmos Phys 63:119-129. doi: 10.1007/Bf01025368

Lubbers IM, van Groenigen JW (2013) A simple and effective method to keep earthworms confined to open-top mesocosms. Appl Soil Ecol 64:190-193. doi: 10.1016/j.apsoil.2012.12.008

Rockel B, Will A, Hense A (2008) The Regional Climate Model COSMO-CLM(CCLM). Meteorol Z 17:347-348. doi: 10.1127/0941-2948/2008/0309

Schädler M, Buscot F, Klotz S, Reitz T, Durka W, Bumberger J, Merbach I, Michalski SG, Kirsch K, Remmler P, Schulz E, Auge H (2019) Investigating the consequences of climate change under different land-use regimes: a novel experimental infrastructure. Ecosphere 10:e02635. doi: 10.1002/ecs2.2635

Singh J, Cameron E, Reitz T, Schädler M, Eisenhauer N (2020) Grassland management effects on earthworm communities under ambient and future climatic conditions. Eur J Soil Sci 72:343-355. doi: 10.1111/ejss.12942

Scheu S (1992) Automated measurement of the respiratory response of soil microcompartments: active microbial biomass in earthworm faeces. Soil Biol Biochem 24:1113-1118. doi: 10.1016/0038-0717(92)90061-2

**
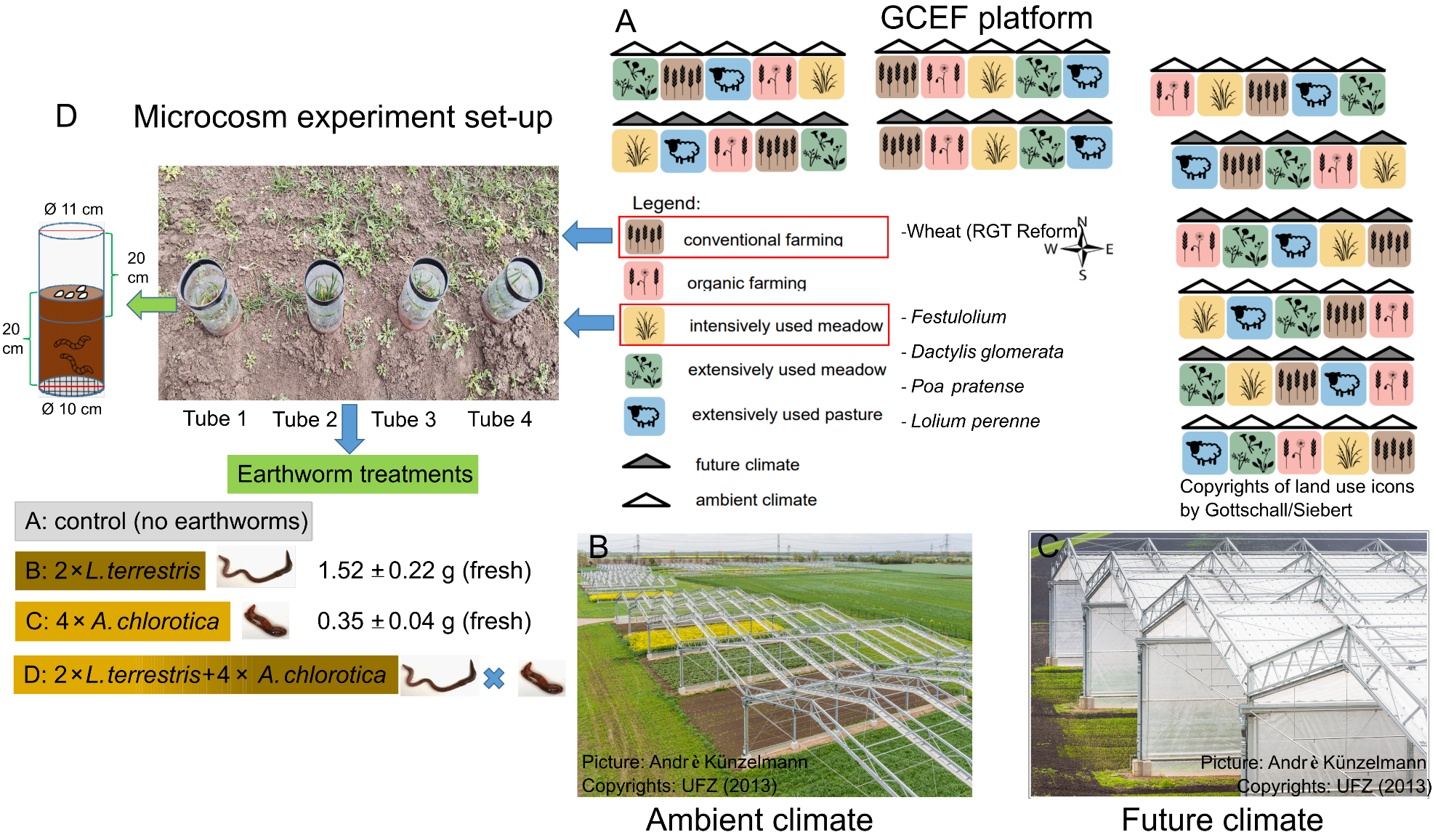
**

**Figure S1.** Global Change Experimental Facility (GCEF) and microcosm experiment set-up.

**Table S1** Timeline of the measures taken, which the wheats, grasses, and earthworms in the concern tubes.

| Date | Action |
| --- | --- |
| 19-Oct-2020 | Sinking of the tubes into the ground |
| 23-Oct-2020 | Sowing the seeds of grass and winter wheat |
| 11-Mar-2021 | Introducing the earthworms in the tubes |
| 22-Mar-2021 | Fertilizer: 60 kg ha^-1^ KAS (27% N) |
| 25-Mar-2021 | Herbicides: 1.5 l ha^-1^Ariane C |
| 07-Apr-2021 | Fertilizer: 40 kg ha^-1^ KAS (27% N) |
| 13-Apr-2021 | Fungicide: 1.5 l ha^-1^ Revystar and 1.5 l ha^-1^ Flexity  Growth regulator: 66.7g kg^-1^ Trinexapac and 42.4g kg^-1^ Prohexadio |
| 15-Apr-2021 | Corn straw (earthworm food): 1 g per tube |
| 05-May-2021 to 06-May-2021 | First grass harvest at a height of 5 cm |
| 06-May-2021 | Corn straw (earthworm food): 0.5 g per tube |
| 17-Jun-2021 to 18-Jun-2021 | Second grass harvest at a height of 5 cm |
| 14-Jul-2021 | Third grass harvest, close to the ground |
| 19-Jul-2021 | Harvesting wheat and grasses roots |

**
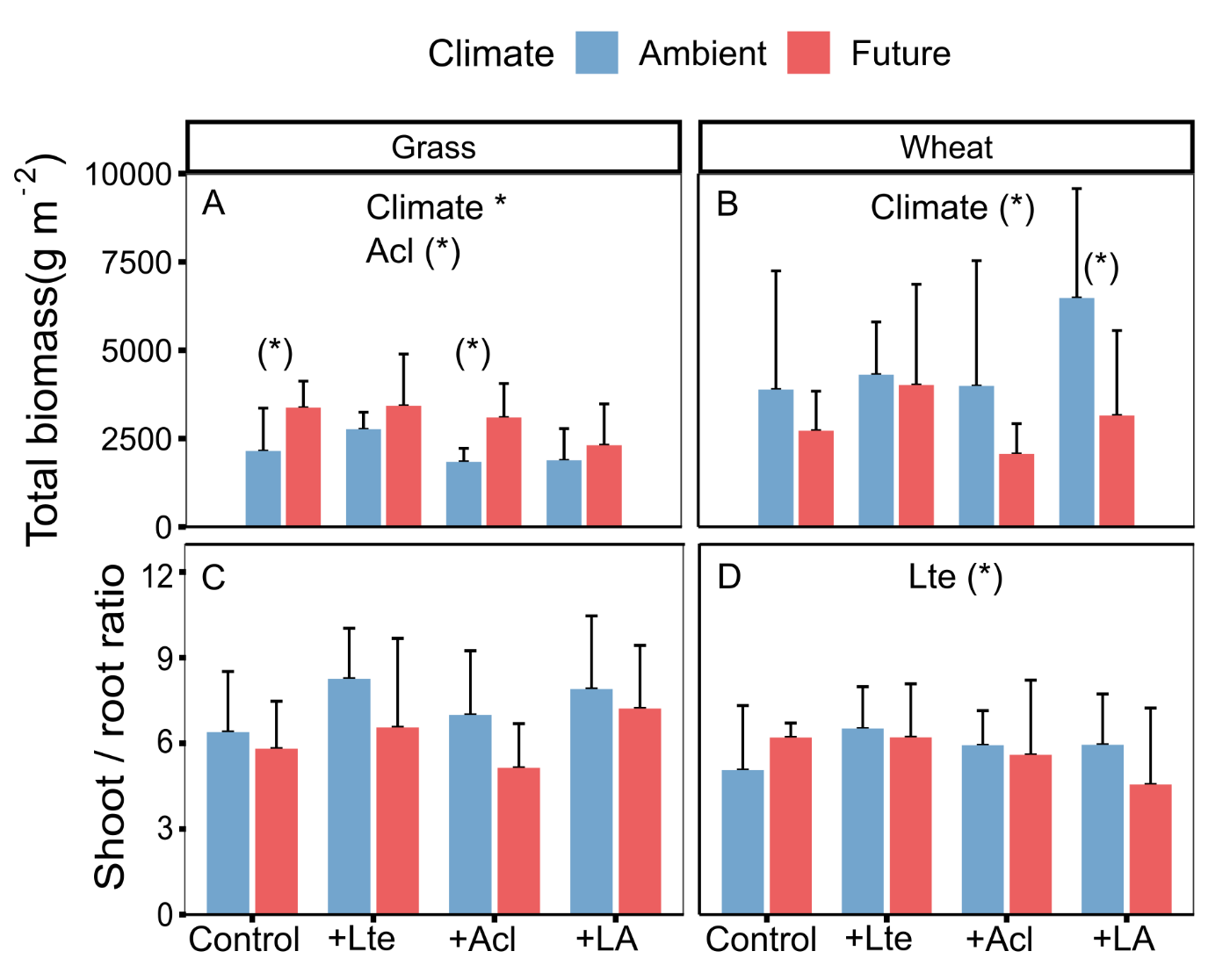
**

**Figure S2.** Effects of climate change and earthworms on total biomass and shoot: root ratio of grasses overall three harvests and total biomass and shoot: root ratio of wheat (mean + *SD*, *N* = 5). Different lowercase letters denote significant (*P* < 0.05) differences among earthworm treatments (Control: no earthworm, +Lte: only with *Lumbricus terrestris*, +Acl: only with *Allolobophora chlorotica*, +LA: mixed *Lumbricus terrestris* and *Allolobophora chlorotica*) based on post-hoc Tukey’s HSD tests. * and (*) denote significant (*P* < 0.05) and marginal (*P* < 0.10) effects of climate, earthworms (Lte: with *Lumbricus terrestris*, Acl: with *Allolobophora chlorotica*), and their interaction based on linear mixed models, as well as significant and marginal differences between climate scenarios based on post-hoc Tukey’s HSD tests, respectively.

**Table S2.** Anova table (*F*-values and significance levels) from linear mixed-effects model testing the effects of climate (C ; ambient vs. future), three times for harvest (H; 05-May-2021, 17-Jun-2021, and 14-Jul-2021), earthworms (Lte: with *Lumbricus terrestris*, Acl: with *Allolobophora chlorotica*), and their interactions on shoot biomass of grass at community and species level (including TotalShoot: total shoot of grasses, Fes: *Festulolium* shoot, Lol: *Lolium perenne* shoot, Dac: *Dactylis glomerata* shoot, Poa: *Poa pratense* shoot) and the shoot mean C content ([C]com), N content ([N]com), and C:N ratio ([CN]com) of grass at community-level. Numerator degree of freedom and denominator degree of freedom were given in column df.

Significant effects are indicated in bold font, with (*)=*P* <0.1, *=*P* < 0.05, **=*P* < 0.01, ***=*P* < 0.001.

|  | *df* | Total shoot | Fes | Lol | Dac | Poa | *df* | [C]com | [N]com | [CN]com |
| --- | --- | --- | --- | --- | --- | --- | --- | --- | --- | --- |
| Climate | 1,8 | **6.63(*)** | **9.46*** | 0.04 | 1.85 | 0.06 | 1 | 0.00 | **8.86*** | 0.69 |
| Harvest | 2,16 | **85.24***** | **86.30***** | **41.42***** | **54.14***** | 1.86 | 2 | **126.27***** | **356.26***** | **174.28***** |
| Lte | 1,24 | 0.00 | 0.03 | 0.30 | **5.04*** | 0.03 | 1 | 0.54 | 1.25 | 0.28 |
| Acl | 1,24 | **4.18(*)** | **3.90(*)** | 0.54 | 0.03 | 1.75 | 1 | 1.30 | 1.80 | 0.24 |
| C × H | 2,16 | **10.28**** | **9.55**** | 0.01 | **8.08**** | 1.31 | 2 | **6.59**** | **37.61***** | **7.84**** |
| C × Lte | 1,24 | 1.10 | 0.90 | 1.81 | 0.52 | 1.30 | 1 | 0.67 | **3.09(*)** | 0.79 |
| C × Acl | 1,24 | 0.02 | 0.16 | 1.45 | 0.37 | 0.05 | 1 | **4.89*** | 2.60 | 0.21 |
| H ×Lte | 2,48 | 0.35 | 0.22 | 0.17 | 1.44 | 0.05 | 2 | **4.23*** | 2.29 | 0.44 |
| H × Acl | 2,48 | **4.11*** | **3.71*** | 0.18 | 0.03 | 2.22 | 2 | **6.82**** | **6.22**** | 2.38 |
| C × H × Lte | 2,48 | 2.31 | 1.91 | **4.19*** | 0.36 | **3.70*** | 2 | 1.73 | **4.35*** | 0.94 |
| C × H × Acl | 2,48 | 0.79 | 0.81 | 0.49 | 0.26 | 0.54 | 2 | **4.86*** | **3.07(*)** | 0.21 |
| Lte × Acl | 1,24 | 1.28 | 0.95 | 0.50 | 0.52 | 0.07 | 1 | 1.18 | 1.32 | 1.43 |
| C × Lte × Acl | 1,24 | 0.16 | 0.15 | 0.82 | 2.41 | 0.38 | 1 | 0.17 | **4.67*** | 0.87 |
| H × Lte × Acl | 2,48 | 0.81 | 0.54 | 0.56 | 1.25 | 1.03 | 2 | 0.16 | 0.84 | 0.58 |
| C × H × Lte × Acl | 2,48 | 0.23 | 0.10 | 0.10 | **2.46(*)** | 0.97 | 2 | 0.44 | **4.01*** | 1.26 |


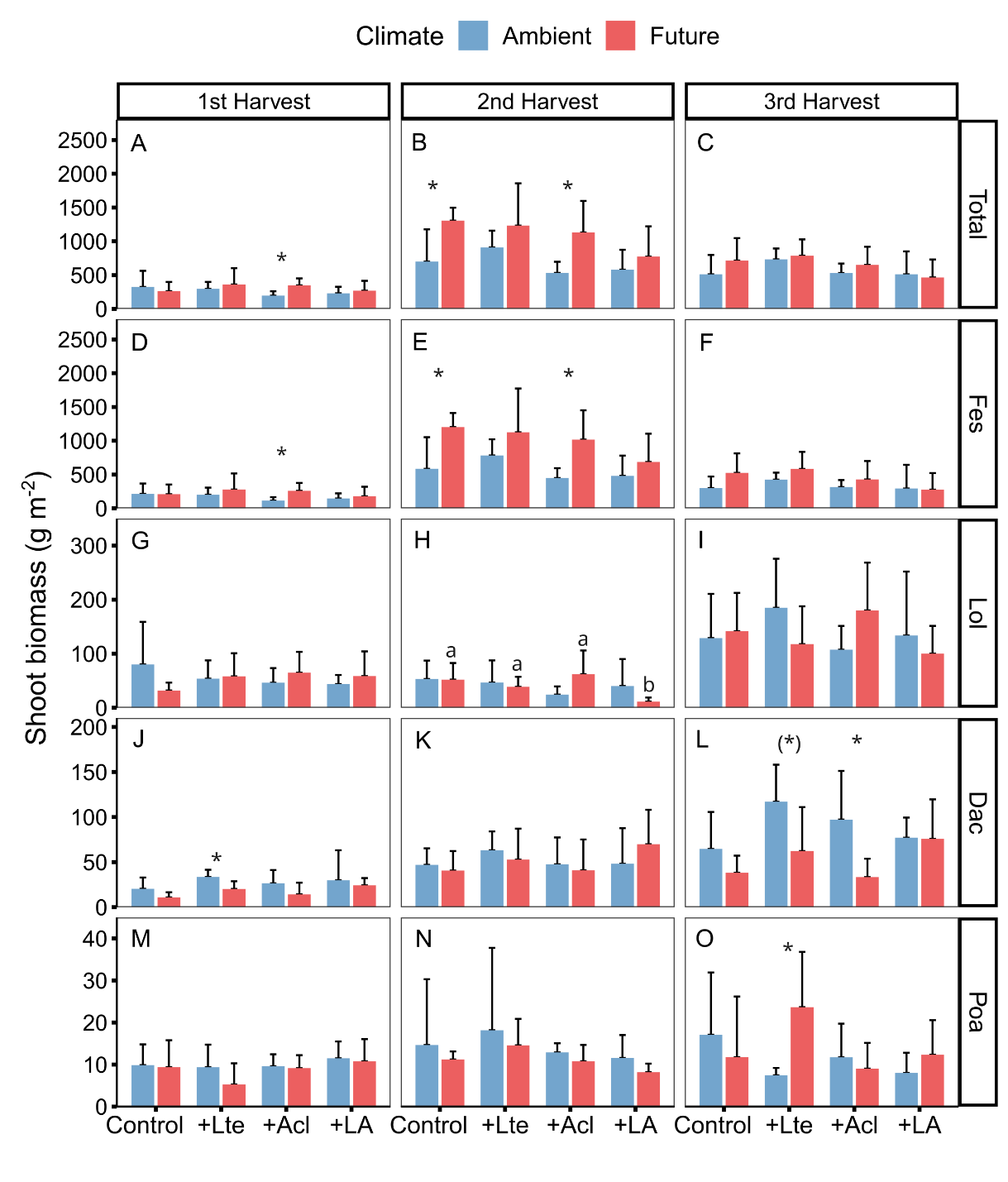


**Figure S3.** Effects of climate change and earthworms on shoot biomass of grass at community and species level (including TotalShoot: total shoot biomass of grass community, *Festulolium*, *Lolium perenne*, *Dactylis glomerata*, and *Poa pratense*) in three harvests (1st harvest = 05-May-2021, 2nd harvest= 17-Jun-2021 and 3rd harvest= 14-Jul-2021; mean + *SD*, *N* = 5). Different lowercase letters denote significant (*P* < 0.05) differences among earthworm treatments (Control: no earthworm, +Lte: only with *Lumbricus terrestris*, +Acl: only with *Allolobophora chlorotica*, +LA: mixed *Lumbricus terrestris* and *Allolobophora chlorotica*) based on post-hoc Tukey’s HSD tests. Asterisks and (*) denote significant (**P* < 0.05, ***P* < 0.01, ****P* < 0.001) and marginal (*P* < 0.10) differences between climate scenarios based on post-hoc Tukey’s HSD tests, respectively.

**
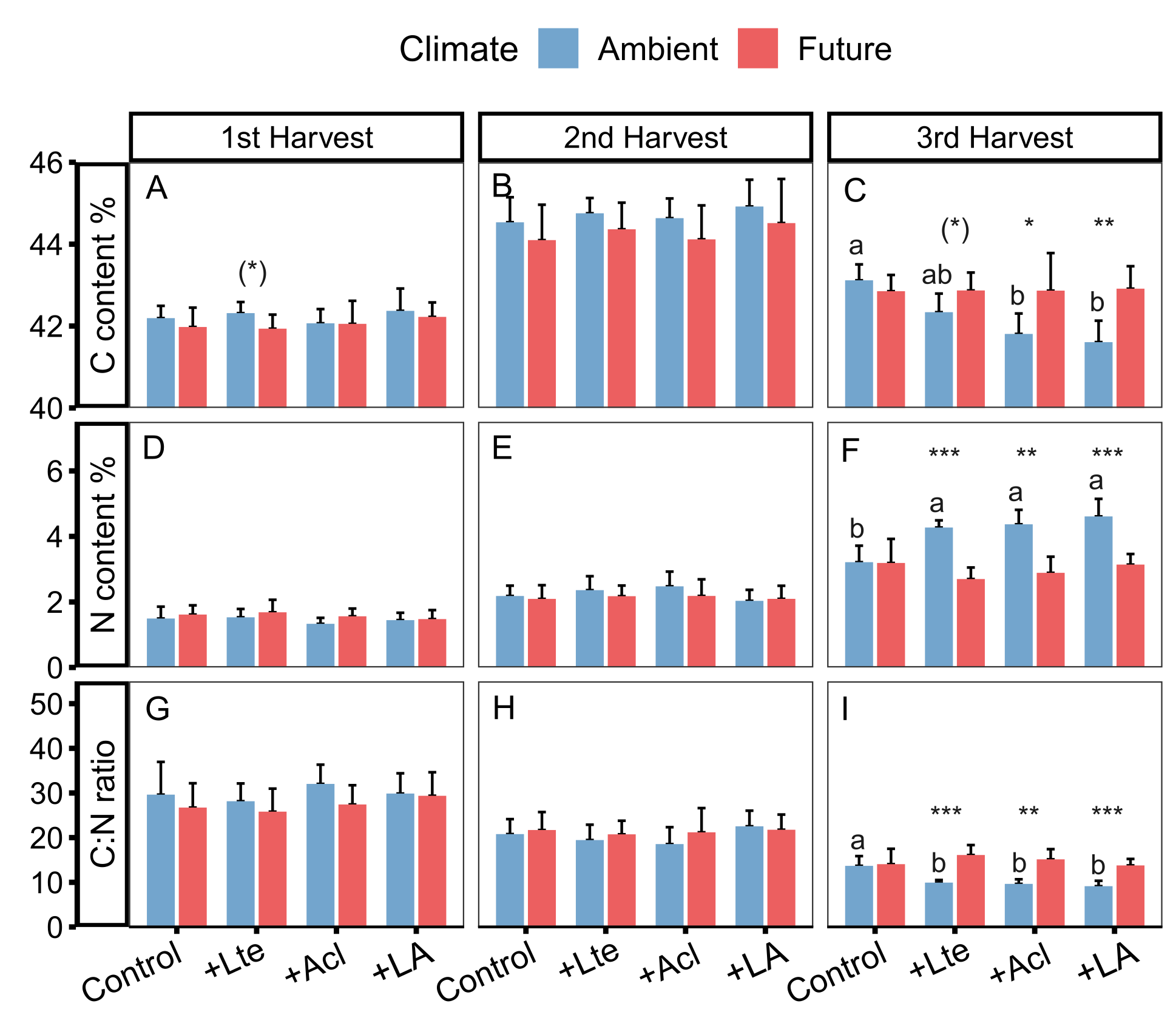
**

**Figure S4.** Effects of climate change and earthworms on the grass community-level mean C content, N content, and C:N ratio in three harvests (1st harvest = 05-May-2021, 2nd harvest= 17-Jun-2021 and 3rd harvest= 14-Jul-2021; mean + *SD*, *N* = 5). Different lowercase letters denote significant (*P* < 0.05) differences among earthworm treatments (Control: no earthworm, +Lte: only with *Lumbricus terrestris*, +Acl: only with *Allolobophora chlorotica*, +LA: mixed *Lumbricus terrestris* and *Allolobophora chlorotica*) based on post-hoc Tukey’s HSD tests. Asterisks and (*) denote significant (**P* < 0.05, ***P* < 0.01, ****P* < 0.001) and marginal (*P* < 0.10) differences between climate scenarios based on post-hoc Tukey’s HSD tests, respectively

**Table S3.** Results (*F*-values and significance levels) from linear mixed-effects models testing the effects of climate (C; ambient vs. future), earthworms (Lte: with *Lumbricus terrestris*, Acl: with *Allolobophora chlorotica*), and their interactions on carbon and nitrogen pool of aboveground biomass of grasses, above- and belowground biomass of wheat. Numerator degree of freedom and denominator degree of freedom were given in the first row.

Significant effects are indicated in bold font, with (*) = *P* < 0.1, *=*P* < 0.05, **=*P* < 0.01, ***=*P* < 0.001.

|  |  | Climate  *df* 1,8 | Lte  *df* 1,24 | Acl  *df* 1,24 | C × Lte  *df* 1,24 | C × Acl  *df* 1,24 | Lte × Acl  *df* 1,24 | C × Lte × Acl  *df* 1,24 |
| --- | --- | --- | --- | --- | --- | --- | --- | --- |
| Grass below | Carbon pool | **14.57**** | 0.51 | **10.23**** | 1.13 | **9.67**** | 1.61 | 0.28 |
|  | Nitrogen pool | 2.67 | 0.06 | 1.24 | 1.25 | 2.75 | 2.54 | 0.04 |
| Wheat above | Carbon pool | **4.05(*)** | 2.49 | 0.02 | 0.68 | 0.89 | 0.63 | 0.02 |
|  | Nitrogen pool | **5.68*** | 1.71 | 0.00 | 0.54 | 0.82 | 0.56 | 0.00 |
| Wheat below | Carbon pool | 0.63 | 0.51 | 0.07 | 1.38 | 1.35 | 0.47 | 2.83 |
|  | Nitrogen pool | 0.22 | 0.03 | 2.92 | 2.65 | 2.17 | 2.01 | **8.24**** |


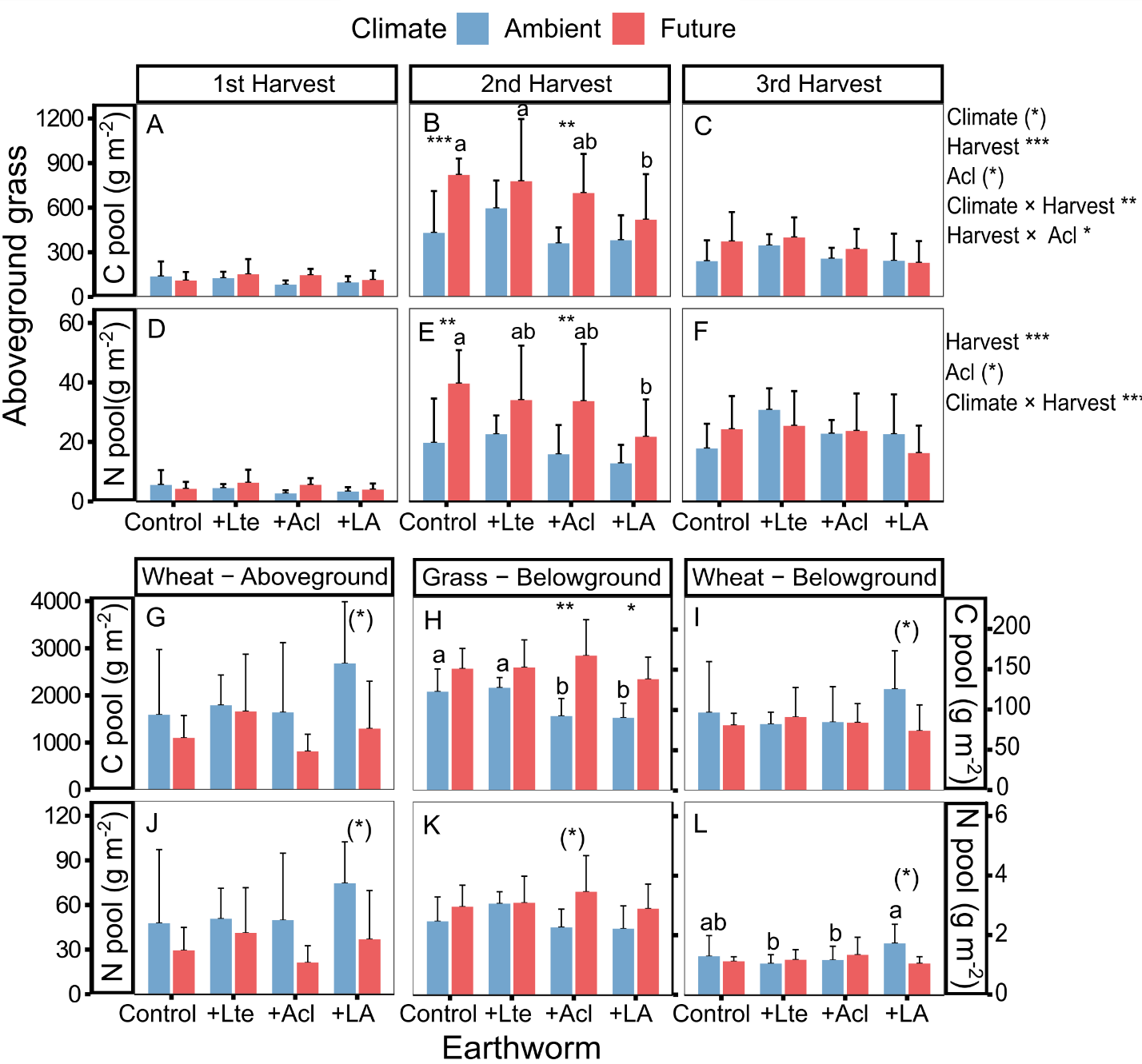


**Figure S5.** Effects of climate change and earthworms on carbon and nitrogen pool of above- and belowground biomass of grasses and wheat (mean + *SD*, *N* = 5). Different lowercase letters denote significant (*P* < 0.05) differences among earthworm treatments (Control: no earthworm, +Lte: only with *Lumbricus terrestris*, +Acl: only with *Allolobophora chlorotica*, +LA: mixed *Lumbricus terrestris* and *Allolobophora chlorotica*) based on post-hoc Tukey’s HSD tests. Asterisks and (*) denote significant (**P* < 0.05, ***P* < 0.01, ****P* < 0.001) and marginal (*P* < 0.10) effects of climate, earthworms (Lte: with *Lumbricus terrestris*, Acl: with *Allolobophora chlorotica*), harvest, and their interaction based on linear mixed-effects models, as well as differences between climate scenarios based on post-hoc Tukey’s HSD tests, respectively.

**Table S4.** Anova table (*F*-values and significance levels) from linear mixed-effects model testing the effects of climate (C; ambient vs. future), three times for harvest (H; 05-May-2021, 17-Jun-2021, and 14-Jul-2021), earthworms (Lte: with *Lumbricus terrestris*, Acl: with *Allolobophora chlorotica*), and their interactions on aboveground carbon content, nitrogen content, and C:N ratio of four grass species (including Fes: *Festulolium,* Lol: *Lolium perenne,* Dac: *Dactylis glomerata*, Poa: *Poa pratense*). Numerator degree of freedom and denominator degree of freedom were given in column df.

Significant effects are indicated in bold font, with (*)=*P*<0.1, *=*P* < 0.05, **=*P* < 0.01, ***=*P* < 0.001.

|  |  | Carbon | | | |  | Nitrogen | | | |  | C:N | | | |
| --- | --- | --- | --- | --- | --- | --- | --- | --- | --- | --- | --- | --- | --- | --- | --- |
|  | *df* | Fes | Lol | Dac | Poa |  | Fes | Lol | Dac | Poa |  | Fes | Lol | Dac | Poa |
| Climate | 1,8 | 0.04 | 0.00 | 0.05 | 0.34 |  | **9.35*** | 3.08 | **16.73*** | 2.92 |  | 0.82 | 0.94 | 3.00 | 1.23 |
| Harvest | 2,16 | **141.57***** | **101.37***** | **66.56***** | **57.37***** |  | **285.88***** | **394.90***** | **175.70***** | **74.84***** |  | **146.90***** | **472.19***** | **83.32***** | **79.45***** |
| Lte | 1,24 | 0.22 | 0.34 | 1.57 | **4.25(*)** |  | 1.40 | 1.81 | 2.08 | **6.26*** |  | 0.47 | 1.16 | 0.93 | **7.70*** |
| Acl | 1,24 | 2.09 | **1.89** | 0.39 | 0.02 |  | 1.11 | 0.04 | **4.51*** | 0.01 |  | 0.72 | **3.67(*)** | 0.87 | 0.21 |
| C × H | 2,16 | **7.39**** | 1.53 | **11.10***** | 1.96 |  | **33.06***** | **29.70***** | **29.04***** | **3.22(*)** |  | **7.06**** | **15.48***** | **5.67*** | 0.94 |
| C × Lte | 1,24 | 0.04 | 0.31 | **3.54(*)** | 0.18 |  | **4.45*** | 0.29 | 0.46 | 2.91 |  | 1.99 | 0.00 | 0.92 | **4.02(*)** |
| C × Acl | 1,24 | **7.14*** | 0.23 | **8.76**** | 0.91 |  | **4.72*** | 0.10 | **3.18(*)** | 1.23 |  | 1.36 | **4.18(*)** | 0.23 | **3.99(*)** |
| H ×Lte | 2,48 | **3.15(*)** | 2.31 | **4.31*** | **5.79**** |  | **3.09(*)** | 0.27 | **2.51(*)** | 0.19 |  | 0.40 | 0.08 | 1.12 | 0.02 |
| H × Acl | 2,48 | **6.37**** | 0.27 | **6.92**** | 0.18 |  | **5.30**** | **3.60*** | **3.24*** | **3.15(*)** |  | 2.01 | 2.37 | 0.23 | 1.31 |
| C × H × Lte | 2,48 | 2.01 | 0.96 | 0.43 | 0.15 |  | **3.76*** | 0.67 | **6.77**** | 0.36 |  | 1.79 | 0.20 | 1.45 | 0.62 |
| C × H × Acl | 2,48 | **6.83**** | 0.07 | **6.22**** | 0.38 |  | **3.75*** | **2.44(*)** | **4.90*** | **2.90(*)** |  | 0.25 | 1.54 | 1.65 | 2.11 |
| Lte × Acl | 1,24 | **3.22(*)** | 1.41 | 2.02 | 0.01 |  | 1.06 | 2.79 | 0.00 | 2.70 |  | 1.11 | 1.07 | 0.01 | 1.47 |
| C × Lte × Acl | 1,24 | 0.20 | 1.70 | 1.19 | 1.84 |  | **4.27*** | 1.23 | **5.83*** | 1.04 |  | 0.58 | 0.12 | **2.97(*)** | 0.03 |
| H × Lte × Acl | 2,48 | 0.65 | 0.68 | 1.52 | 0.18 |  | 0.68 | 0.35 | 0.80 | 1.11 |  | 0.48 | 0.11 | 0.83 | 0.15 |
| C × H × Lte × Acl | 2,48 | 0.47 | 0.64 | 1.67 | 0.04 |  | **4.91*** | 0.74 | **5.94**** | **6.35**** |  | 1.50 | 0.19 | **5.40**** | 1.87 |


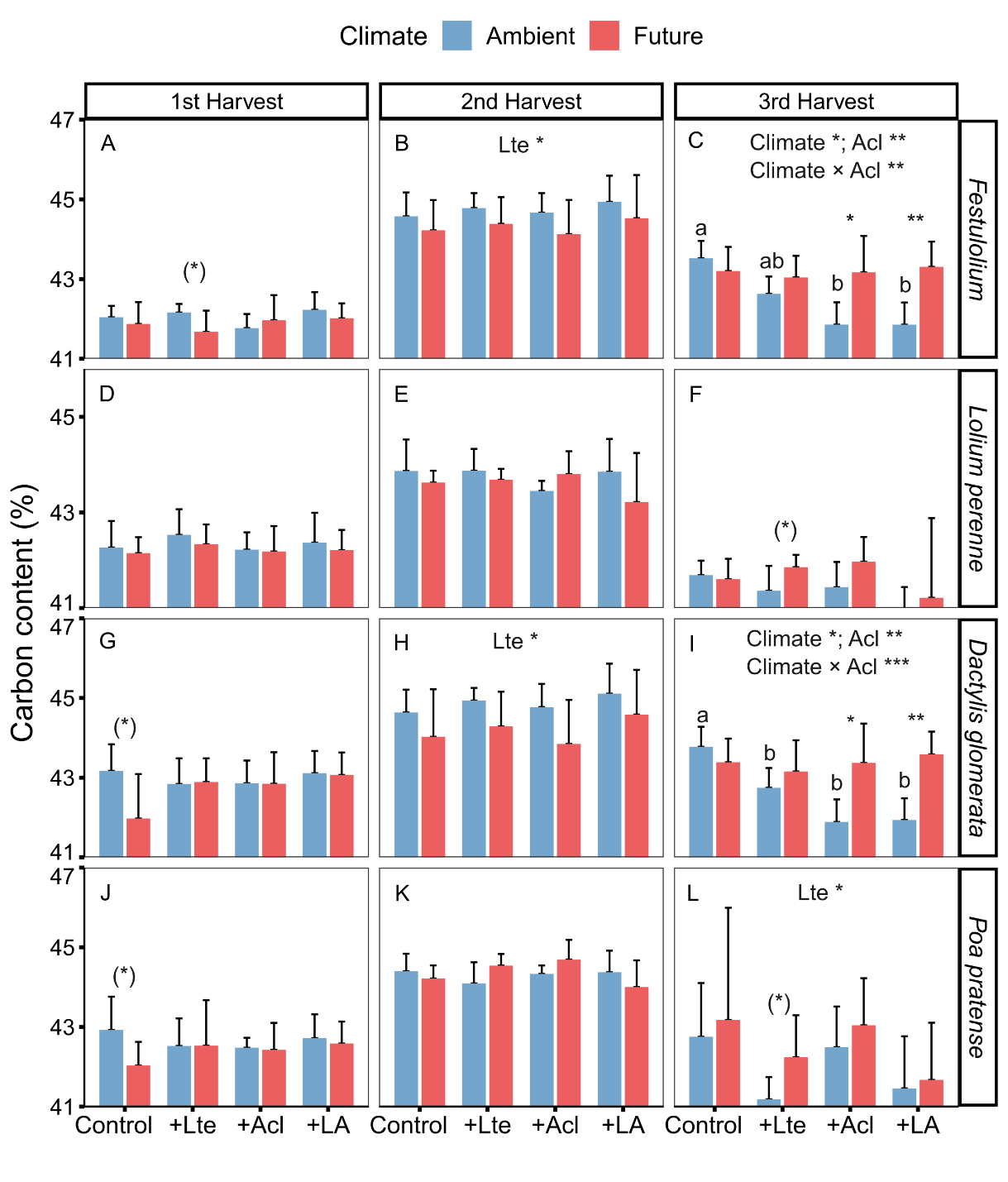


**Figure S6.** Effects of climate change and earthworms on aboveground carbon content of four grass species (including, *Festulolium*, *Lolium perenne*, *Dactylis glomerata*, and *Poa pratense*) in three harvests (1st harvest = 05-May-2021, 2nd harvest= 17-Jun-2021 and 3rd harvest= 14-Jul-2021; mean + *SD*, *N* = 5). Different lowercase letters denote significant (*P* < 0.05) differences among earthworm treatments (Control: no earthworm, +Lte: only with *Lumbricus terrestris*, +Acl: only with *Allolobophora chlorotica*, +LA: mixed *Lumbricus terrestris* and *Allolobophora chlorotica*) based on post-hoc Tukey’s HSD tests. Asterisks and (*) denote significant (**P* < 0.05, ***P* < 0.01, ****P* < 0.001) and marginal (*P* < 0.10) differences between climate scenarios based on post-hoc Tukey’s HSD tests, respectively.


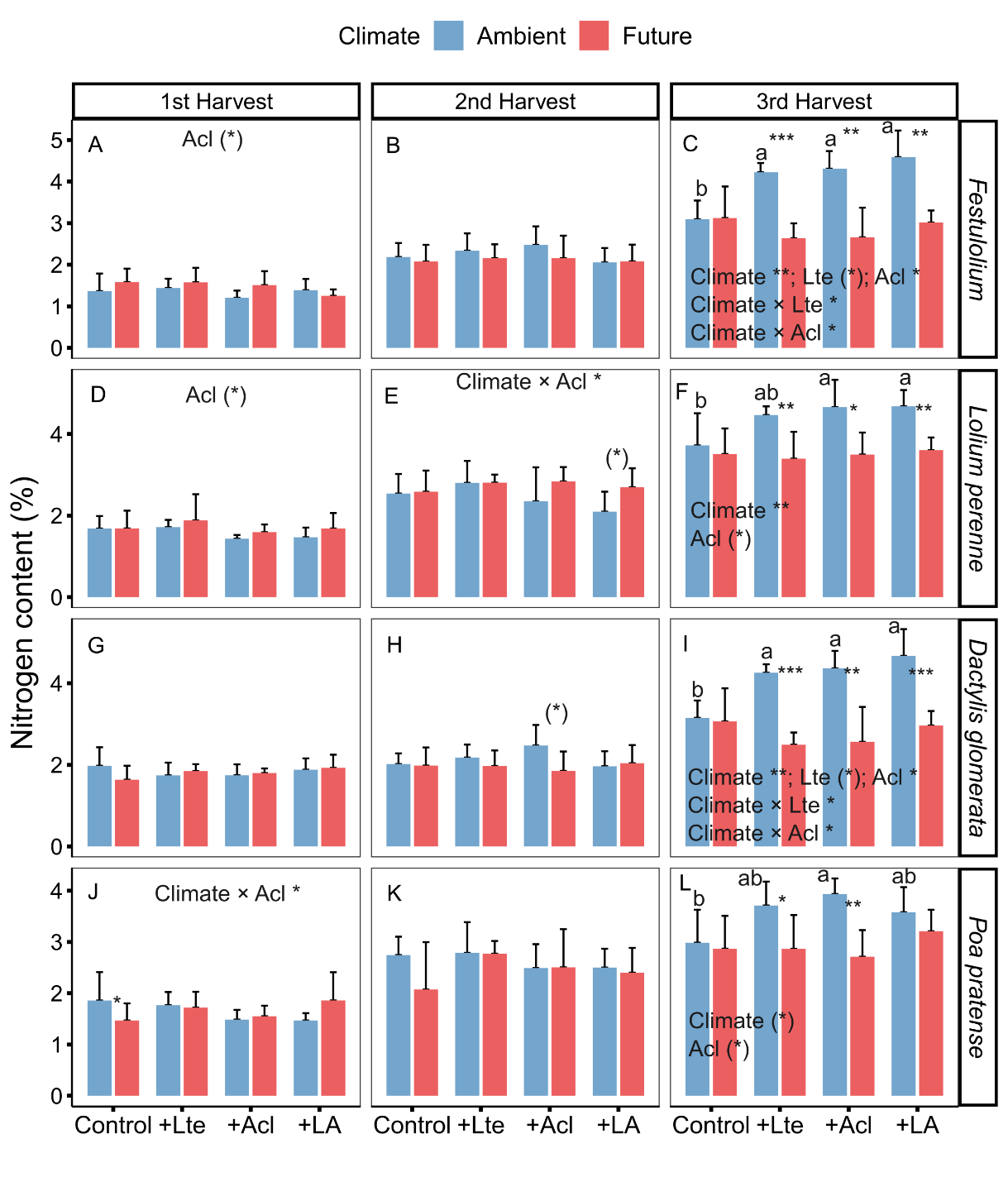


**Figure S7.** Effects of climate change and earthworms on aboveground nitrogen content of four grass species (including, *Festulolium*, *Lolium perenne*, *Dactylis glomerata*, and *Poa pratense*) in three harvests (1st harvest = 05-May-2021, 2nd harvest= 17-Jun-2021 and 3rd harvest= 14-Jul-2021; mean + *SD*, *N* = 5). Different lowercase letters denote significant (*P* < 0.05) differences among earthworm treatments (Control: no earthworm, +Lte: only with *Lumbricus terrestris*, +Acl: only with *Allolobophora chlorotica*, +LA: mixed *Lumbricus terrestris* and *Allolobophora chlorotica*) based on post-hoc Tukey’s HSD tests. Asterisks and (*) denote significant (**P* < 0.05, ***P* < 0.01, ****P* < 0.001) and marginal (*P* < 0.10) differences between climate scenarios based on post-hoc Tukey’s HSD tests, respectively.


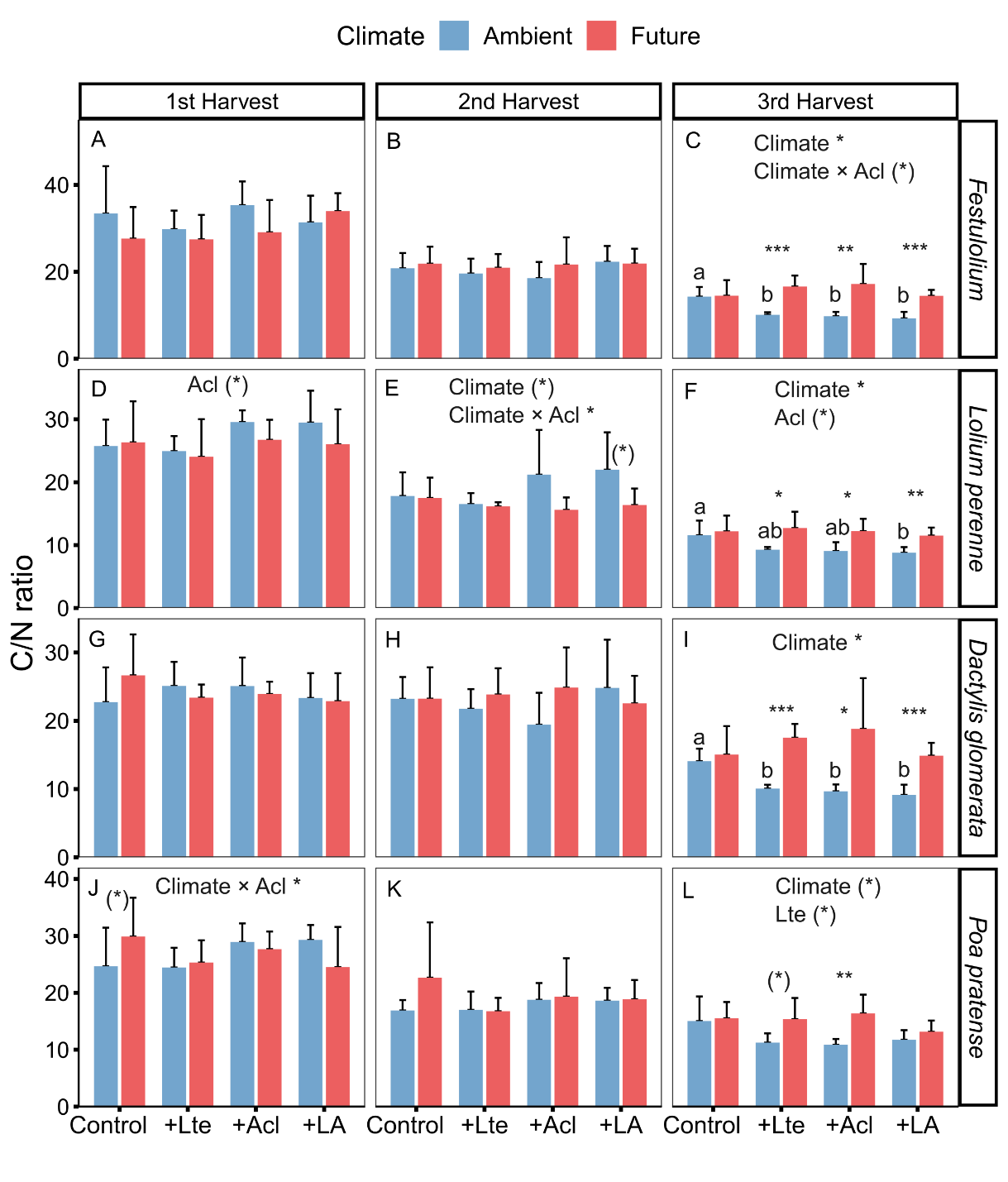


**Figure S8.** Effects of climate change and earthworms on aboveground C:N ratio of four grass species (including, *Festulolium*, *Lolium perenne*, *Dactylis glomerata*, and *Poa pratense*) in three harvests (1st harvest = 05-May-2021, 2nd harvest= 17-Jun-2021 and 3rd harvest= 14-Jul-2021; mean + *SD*, *N* = 5). Different lowercase letters denote significant (*P* < 0.05) differences among earthworm treatments (Control: no earthworm, +Lte: only with *Lumbricus terrestris*, +Acl: only with *Allolobophora chlorotica*, +LA: mixed *Lumbricus terrestris* and *Allolobophora chlorotica*) based on post-hoc Tukey’s HSD tests. Asterisks and (*) denote significant (**P* < 0.05, ***P* < 0.01, ****P* < 0.001) and marginal (*P* < 0.10) differences between climate scenarios based on post-hoc Tukey’s HSD tests, respectively.

**Table S5.** Results (*F*-values and significance levels) from linear mixed-effects models testing the effects of climate (C; ambient vs. future), earthworms (Lte: with *Lumbricus terrestris*, Acl: with *Allolobophora chlorotica*), and their interactions on biomass, carbon content, nitrogen content and C:N ratio of wheat spike and wheat straw. Numerator degree of freedom and denominator degree of freedom were given in the first row.

Significant effects are indicated in bold font, with (*) = *P* < 0.1, *=*P* < 0.05, **=*P* < 0.01, ***=*P* < 0.001.

|  |  | Climate  *df* 1,8 | Lte  *df* 1,24 | Acl  *df* 1,24 | C × Lte  *df* 1,24 | C × Acl  *df* 1,24 | Lte × Acl  *df* 1,24 | C × Lte × Acl  *df* 1,24 |
| --- | --- | --- | --- | --- | --- | --- | --- | --- |
| Wheat spike | Biomass | 3.19 | **3.63(*)** | 1.17 | 0.70 | 0.56 | 0.41 | 0.00 |
|  | Carbon | 2.32 | 1.37 | 0.21 | 0.03 | 1.28 | 2.22 | 0.31 |
|  | Nitrogen | 1.08 | 1.43 | 2.00 | 0.07 | 0.23 | 0.07 | 0.36 |
|  | C/N ratio | 1.49 | 1.24 | 1.89 | 0.02 | 0.26 | 0.02 | 0.59 |
| Wheat straw | Biomass | 1.02 | 2.49 | 0.05 | 1.01 | 0.04 | 0.17 | 0.00 |
|  | Carbon | 1.54 | **4.47*** | 0.49 | 0.43 | 1.00 | 1.27 | 0.65 |
|  | Nitrogen | 0.43 | 0.50 | **5.47*** | 0.03 | 1.24 | 0.09 | 0.00 |
|  | C/N ratio | 0.95 | 0.24 | **7.24**** | 0.04 | 1.09 | 0.15 | 0.05 |


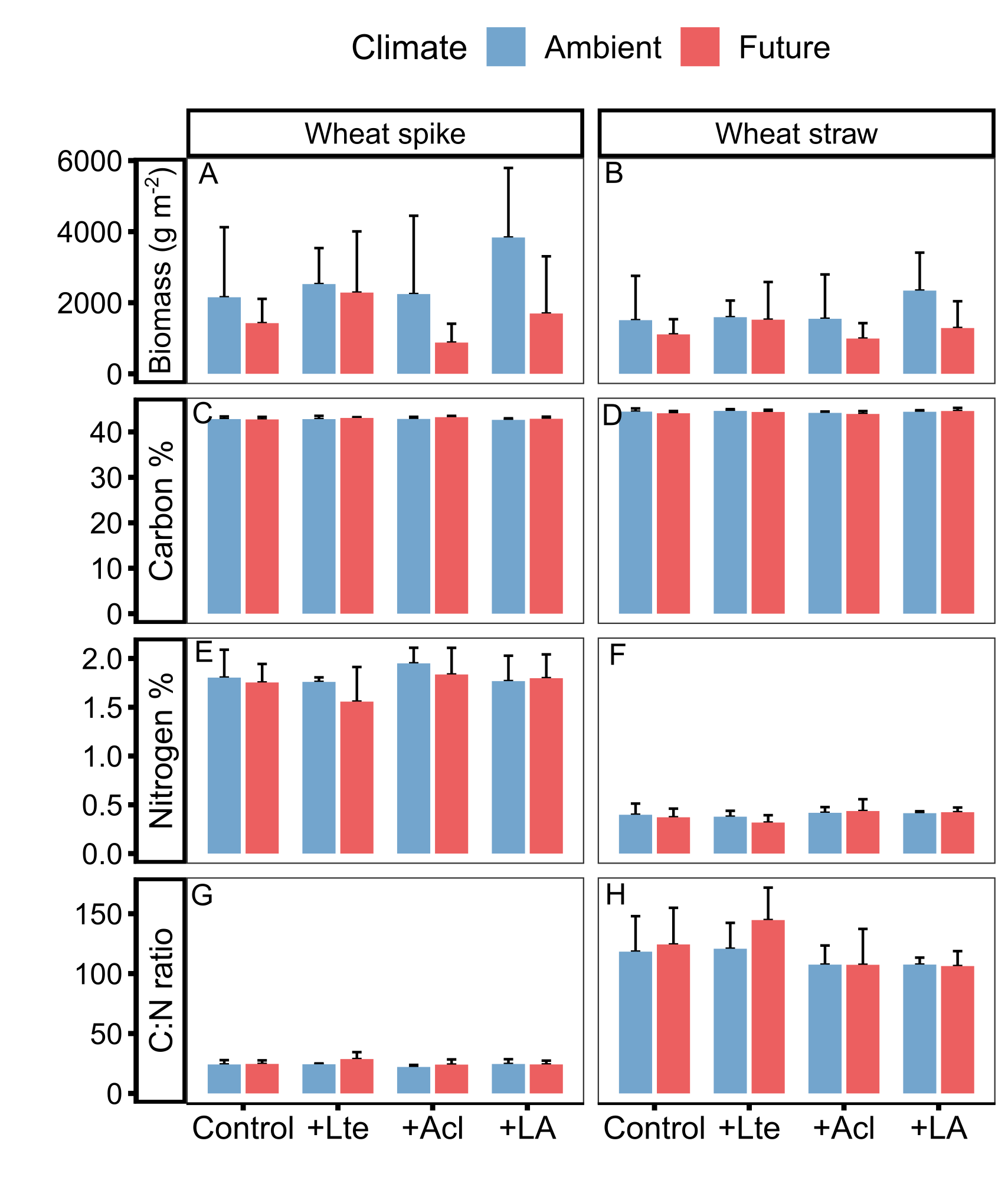


**Figure S9.** Effects of climate change and earthworms on biomass, carbon, nitrogen, and C:N ratio of wheat spike and wheat straw (mean + *SD*, *N* = 5). Different lowercase letters denote significant (*P* < 0.05) differences among earthworm treatments (Control: no earthworm, +Lte: only with *Lumbricus terrestris*, +Acl: only with *Allolobophora chlorotica*, +LA: mixed *Lumbricus terrestris* and *Allolobophora chlorotica*) based on post-hoc Tukey’s HSD tests. Asterisks and (*) denote significant (**P* < 0.05, ***P* < 0.01, ****P* < 0.001) and marginal (*P* < 0.10) differences between climate scenarios based on post-hoc Tukey’s HSD tests, respectively.


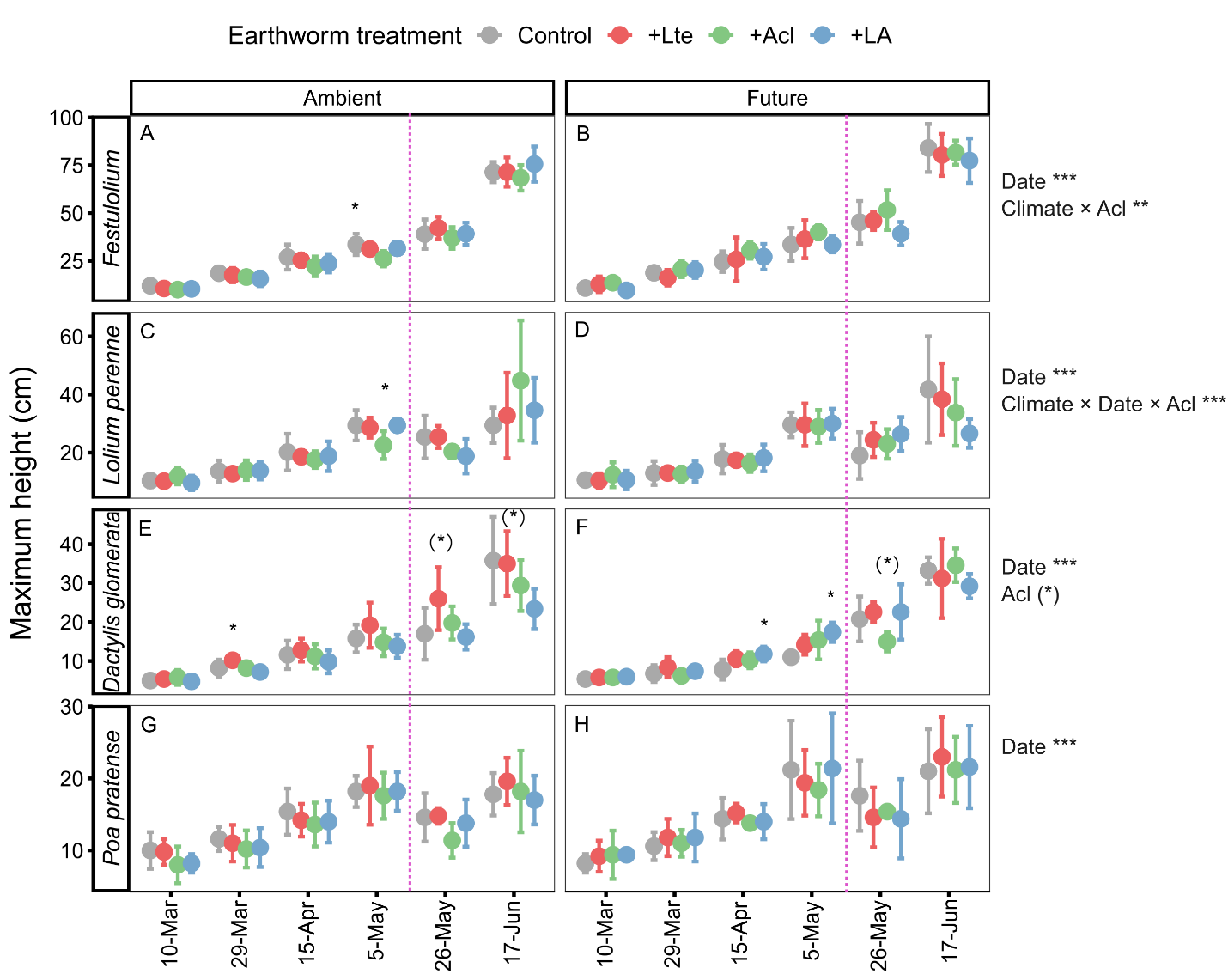


**Figure S10.** Effects of climate change and earthworms on maximum height of grasses from 10-Mar to 17-Jul (mean + *SD*, *N* = 5). * and (*) denote significant (*P* < 0.05) and marginal (*P* < 0.10) effects of climate, earthworms (Lte: with *Lumbricus terrestris*, Acl: with *Allolobophora chlorotica*), measuring date and their interaction based on linear mixed-effects models, as well as significant and marginal differences among earthworm treatments (Control: no earthworm, +Lte: only with *Lumbricus terrestris*, +Acl: only with *Allolobophora chlorotica*, +LA: mixed *Lumbricus terrestris* and *Allolobophora chlorotica*) based on post-hoc Tukey’s HSD tests, respectively.


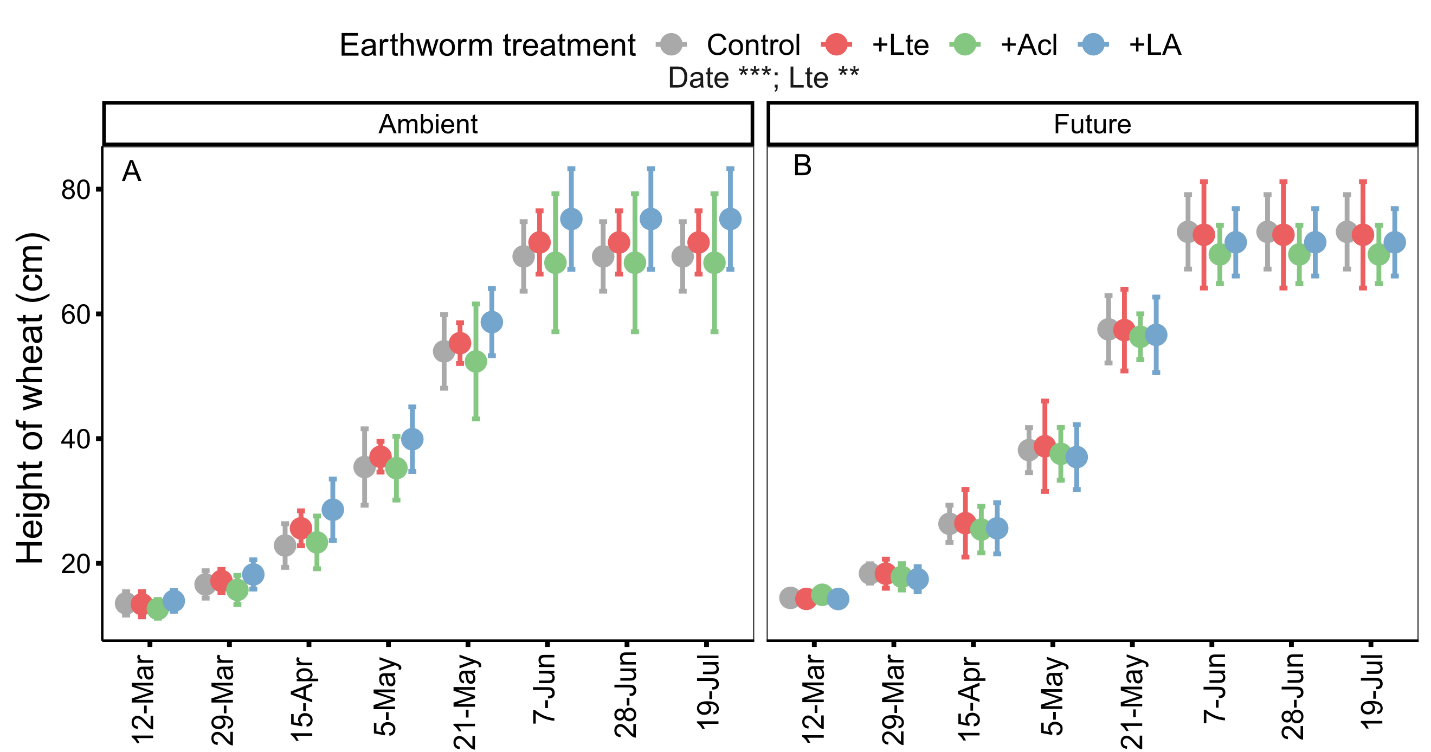


**Figure S11.** Effects of climate change and earthworms on maximum height of wheat from 12-Mar to 19-Jul (mean + *SD*, *N* = 5). * and (*) denote significant (*P* < 0.05) and marginal (*P* < 0.10) of climate, earthworms (Lte: with *Lumbricus terrestris*, Acl: with *Allolobophora chlorotica*), measuring date and their interaction based on linear mixed-effects models, as well as significant and marginal differences among earthworm treatments (Control: no earthworm, +Lte: only with *Lumbricus terrestris*, +Acl: only with *Allolobophora chlorotica*, +LA: mixed *Lumbricus terrestris* and *Allolobophora chlorotica*) based on post-hoc Tukey’s HSD tests, respectively.


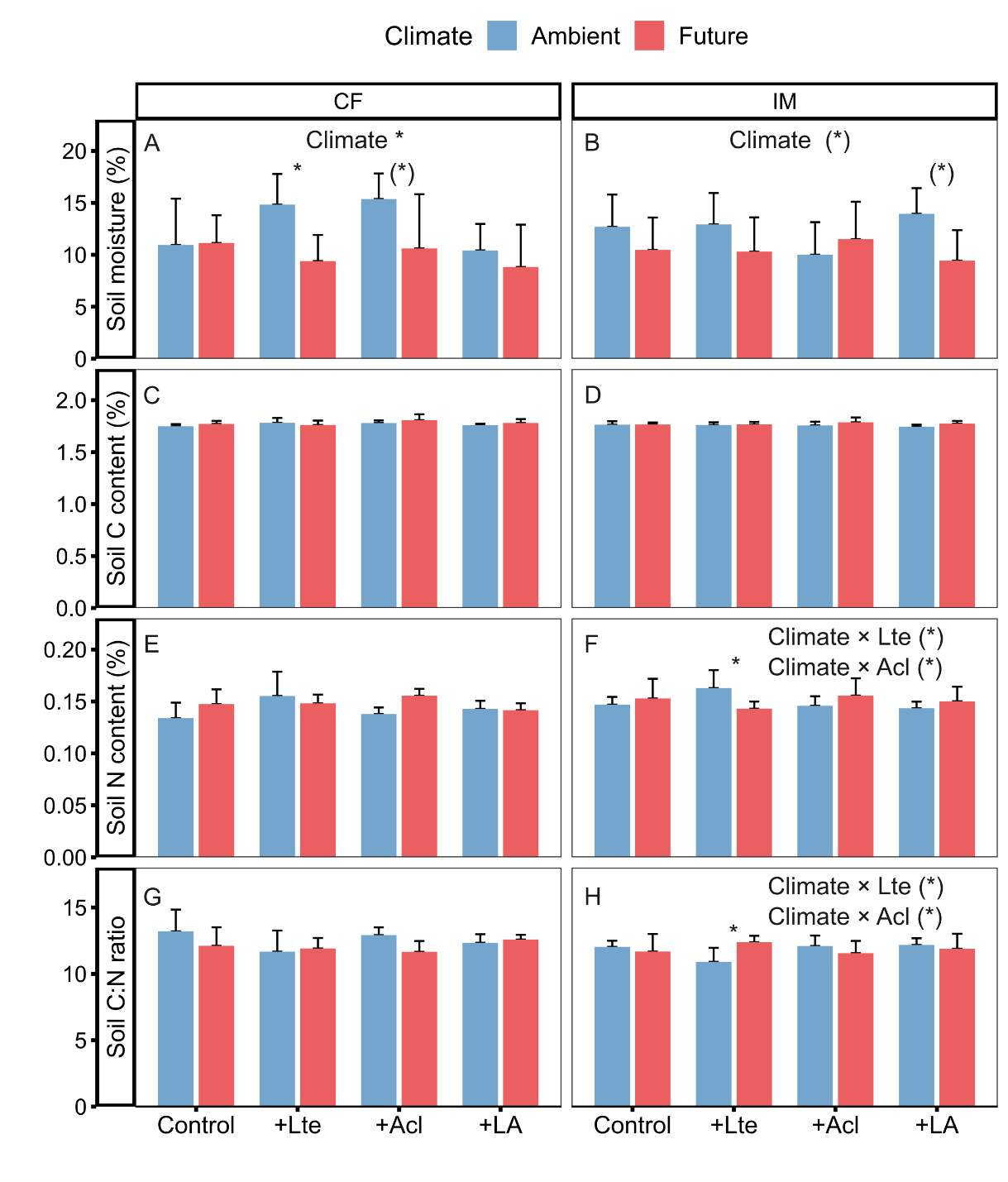


**Figure S12.** Soil moisture, soil C content, soil N content, and Soil C:N ratio in conventional farming (CF) and intensively used meadow (IM) in July (mean + *SD*, *N* = 5). * and (*) denote significant (*P* < 0.05) and marginal (*P* < 0.10) of climate, earthworms (Lte: with *Lumbricus terrestris*, Acl: with *Allolobophora chlorotica*), and their interaction based on linear mixed-effects models, as well as significant and marginal differences between climate scenarios based on post-hoc Tukey’s HSD tests, respectively. Different lowercase letters denote significant (*P* < 0.05) differences among earthworm treatments (Control: no earthworm, +Lte: only with *Lumbricus terrestris*, +Acl: only with *Allolobophora chlorotica*, +LA: mixed *Lumbricus terrestris* and *Allolobophora chlorotica*) based on post-hoc Tukey’s HSD tests.


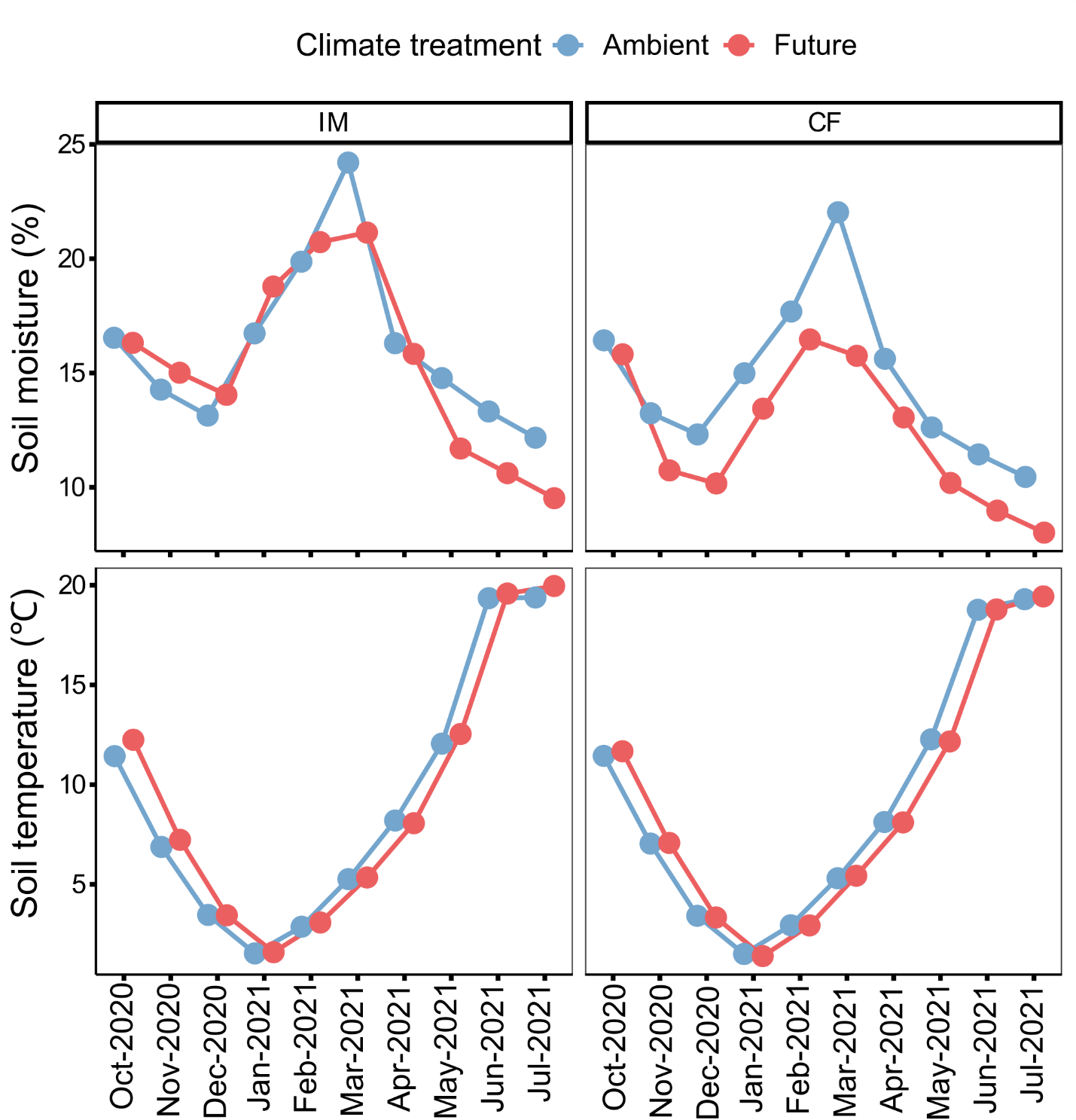


**Figure S13.** Monthly mean soil moisture and soil temperature under ambient and future climate scenarios in conventional farming (CF) and intensively used meadow (IM) at 15 cm soil depths during the period of this experiment.
